# Strengthening capacity for natural sciences research: A qualitative assessment to identify good practices, capacity gaps and investment priorities in African research institutions

**DOI:** 10.1371/journal.pone.0228261

**Published:** 2020-01-24

**Authors:** Taghreed El Hajj, Stefanie Gregorius, Justin Pulford, Imelda Bates

**Affiliations:** 1 Centre for Capacity Research, Liverpool School of Tropical Medicine, Liverpool, England, United Kingdom; 2 Gesellschaft für Internationale Zusammenarbeit (GIZ), Bonn; Universitat Luzern, SWITZERLAND

## Abstract

**Background:**

Strengthening research capacity in low-and-middle-income countries is essential to drive socioeconomic development and to achieve the Sustainable Development Goals. Understanding strengths and weaknesses in institutions’ research capacity can guide effective targeting of investments and resources. This study assessed the capacity of institutions undertaking research in natural science topics in Africa to identify priority capacity gaps for future investment.

**Methods:**

Assessments were conducted in eight African institutions that were partners in a UK-Africa programme to strengthen research capacity in renewable energy, soil-related science, and water and sanitation. Assessments involved eighty-six interviews and three focus group discussions to identify institutions’ research capacity strengths and gaps against an evidence-informed benchmark. Use of the same interview guides and data collection processes across all institutions meant that findings could be compared.

**Results:**

Common research capacity gaps were: lack of, or poorly maintained, equipment; unreliable, slow procurement systems; insufficient opportunities for developing the skills of research support staff such as administrators and technicians; dysfunctional institutional email communication systems; insufficient focus on the development of ‘soft’ researcher skills such as ethics, academic writing and, in non-Anglophone countries, English language. Programme strengths were the South-South and South-North partnerships for sharing and cascading expertise and resources, joint writing of proposals and publications, and improved individual and institutional visibility.

**Conclusion:**

There were many similarities in research capacity gaps irrespective of the institutions’ natural sciences research focus, and these were similar to those reported in the health sector. Common capacity needs are improving the skills of technicians and administrators to support research activities, soft skills training for researchers, and more effective pan-institutional e-communication systems. These could be strategic investment targets for the joint efforts of national governments and international organisations that fund programmes for strengthening research capacity in low- and middle-income countries.

## Background

Strengthening research capacity in poorer countries is a goal of many international research funders and development agencies based on the notion that investment in research can solve problems, overcome challenges and thereby drive socioeconomic development [1]. For example, one of these problems could be lack of access to sustainable energy. One of the sustainable development goals (SDGs) is “to ensure access to reliable, affordable, sustainable, and modern energy to all” [2, p.1] due to the realisation of its substantial importance in backing and maintain various sectors such as health, agriculture, education, technology, business, etc. [2]. Innovative research in that field of efficient energy would contribute to economic growth at the national level [3]. Strong research institutions and skilled researchers are essential for low-and-middle-income countries (LMIC) to generate evidence for their own policies [4–5] and to make progress in achieving the Sustainable Development Goals [6]. For example, a study that assessed 30 health research programmes approaches in Ghana concluded that locally led, demand-driven health research is more successful in translating research into action and in responding to the national needs in low-income countries [7]. Yet, research institutions in low-and-middle-income countries, particularly in Africa, face serious challenges in terms of research funding as they do not receive sufficient funds from their governments and they mostly depend on foreign resources [8]. The UK government has committed to increase its investment in science and technology in poorer countries and to use rigorous evaluations of the effectiveness of this investment to drive improvements and reform across the global aid system [9]. Traditionally there has been an assumption that research capacity will automatically grow as an indirect result of carrying out research projects, but it is becoming apparent that this will not happen unless explicit activities [10] to strengthen capacity are included in research programmes, and relevant metrics for outputs and outcomes are included in programme evaluations. The desire to strengthen research capacity as a mainstream, rather than a ‘bolt-on’, activity within programmes has been translated into action through, for example, the Global Challenges Research Fund Growing Capability [11] programme which primarily aims to improve research capacity as well as conduct research.

Although the average growth rate of production of scientific research in Africa is faster than that of the world as a whole [10], the African countries as a whole only produced 2.6% of the world’s total scientific output in 2014 [12] which is around 29 publications per million inhabitants [12]. This reflects the small numbers of researchers in Africa and decades of under-investment in research institutions. Most countries in sub-Saharan Africa have less than 500 researchers (of all disciplines) per million inhabitants compared to over 4000 per million inhabitants in the UK and North America [13]. There are numerous disincentives to pursuing a research career in many African countries. These include heavy teaching loads, weak organisational research systems, lack of national research leadership, limited access to scientific information, slow internet connections and inadequate physical facilities including libraries and laboratories [14] and scarce funding for research by the African governments [7]. Researchers need a conducive environment in which to flourish so some funders are streamlining their research capacity strengthening efforts into large transformative initiatives to strengthen national and institutional research systems, structures, governance and management [15–16].

To inform future global development strategies [10], it is essential to understand which research capacity strengthening activities work best and in what contexts, to be clear about what outcomes these activities are trying to achieve and to make sure these outcomes are realistic [10]. However, despite calls for more robust evaluations of capacity development [17], the evidence needed to inform effective implementation and evaluation of programmes for strengthening research capacity remains weak [18–19]. Measuring the impact of research capacity strengthening programmes is notoriously difficult and the lack of clearly defined goals, assessments or baselines against which to evaluate the success of research capacity strengthening programmes makes it difficult to track their progress and impact [20]. The use of different methods and tools for data collection across programmes mean that comparisons cannot be made and so lessons cannot be learnt to improve and compare future research capacity strengthening programmes.

The UK Government’s Department for International Development (DFID), in partnership with the UK’s Royal Society (RS), has funded the Africa Capacity Building Initiative (ACBI), a pilot study, to ‘strengthen the research and training capacity of higher education institutions and support the development of individual scientists in sub-Saharan Africa through UK-Africa research collaborations’ [21]. Research in the ACBI focuses on three areas—water and sanitation, renewable energy and soil-related science—and aims to initiate lasting improvements in the research environment within the African host institutions. The ACBI comprises ten research consortia; each consortium comprises one UK and three African institutions. Consortia were selected through a competitive process over two rounds of funding in 2015 and 2016. Research analysing the selection process for consortia has been published elsewhere [22].

Twenty-Six African institutions across eighteen sub-Saharan African countries are represented in the ten consortia (S1 Supplementary File). Each consortium receives funding for five years to cover research expenses, travel and subsistence costs, training for PhD students and a contribution to equipment costs. Each consortium is led by a UK-based researcher, who has overall responsibility for the financial management and outputs of their consortium. Of the ten consortia, one focuses solely on water and sanitation, four focus on renewable energy and two focus on soil-related research. The remaining three consortia each focus on more than one research area: one covers soil-related research with water and sanitation, and two cover renewable energy with water and sanitation.

The objective of the current study was to assess the research capacity of African institutions collaborating in the ACBI programme, to identify common capacity gaps that could be good value-for-money multi-institutional investments, and to highlight examples of good practice and problem-solving strategies that could be shared within and beyond the programme. An objective of the ACBI programme is to ‘support science research institutions in Africa to achieve international competitiveness in research and research training’. The capacity assessments were carried out against an evidence-informed benchmark, minimally adapted from one used to assess African institutions’ capacity to undertake health research [23] which covered doctoral training programmes and institutional research support systems.

## Methods

### Theoretical framework

To conduct the research capacity assessments, the research team from the Centre for Capacity Research (CCR) used a benchmark which had been developed for assessing institutional capacity for health research in sub-Saharan Africa. In the current study, a ‘research institution’ was defined as the university department or stand-alone higher education institution. Minor adaptations were needed to make sure that the benchmark was appropriate for natural sciences, rather than health-research [23] and that it aligned with the ACBI programme’s Theory of Change (S2 Supplementary File). Details of the development, contents and use of the benchmark have been published elsewhere [23]. The benchmark aimed to address the main institutional capacity areas needed to provide “optimal academic, administrative and financial support for operational research activities” from the viewpoints of the dean or other senior institutional authority (e.g. provost, vice chancellor, or principal of the institution), faculty research support staff (including laboratory technicians), researchers (including principal investigators, and co-principal investigators), and PhD students [23, p. 3]. Briefly, the benchmark was developed in two parts which were amalgamated for this study. The first part focused on institutional capacity for doctoral programmes [24] and the second on research support systems in African institutions [23]. The benchmark was based on peer-reviewed publications and grey literature such as guidelines and standards. From these documents, all the items pertaining to global good practice standards for international quality PhD programmes [24] and institutional research support systems [23] were used to create a checklist. In the original benchmark for PhD programmes, items were grouped into four categories: institutional policies and structures, research environment and infrastructure, the doctoral programme life cycle and the student experience. For the research support systems benchmark, components were grouped into eight themes: research infrastructure; research skills training, learning and teaching; organisation and management of the institution; supervision and mentorship for staff and students within the institution; financial management and funding; human resources; collaborations and partnerships; and research uptake and sustainability. For the current study the items in the two benchmarks were amalgamated and categorised into six themes: infrastructure and facilities, teaching and learning, research strategies and support, PhD programmes, research financial management and funding, and research collaborations. These themes were merged after the research team has pilot-tested the original assessment tool during one of the site visits and found that some areas were irrelevant to this study or could be grouped together.

### Participants’ recruitment

In order to achieve the objectives of the study we needed to select participants who were knowledgeable about their institutions’ research capacity as to be included as interviewees. Participants therefore included ACBI-affiliated principal investigators or co-principal investigators, PhD students, PhD supervisors, and other key informants. Interviews were carried out face to face during site visits to African research institutions or when participants were visiting the UK, or else virtually. It was not feasible to visit all twenty-six African research institutions involved in the ACBI programme so the CCR research team planned to visit at least one African partner’s institution in each of the ten consortia. When possible, these visits were designed to coincide with consortium-wide meetings (e.g. inception meetings, training workshops) as each consortium carried out at least one consortium meeting per year, hosted by one of the three African research institutions. These events offered a good opportunity for the CCR research team to interact, face-to-face, and engage with a wide range of consortia members from all institutions within a consortium and to conduct a thorough assessment study at the host institution.

Therefore, the selection of these institutions was based on purposive sampling [25–26]. The research team mapped out consortium-meeting activities across all ten consortia and selected site visits in consultation and coordination with the principal investigators. The principal investigators then liaised with relevant stakeholders in their African institution to inform them about the visit and to invite them to be interviewed for the study as key informants. Relevant stakeholders (e.g. provost, vice chancellor, head of department, head of IT, laboratory manager, other research support staff) were recruited because of their position in the institution and their in-depth knowledge of the topic areas to be covered in the data collection. In many cases there was only one person suitable to be interviewed in relation to a specific area (e.g. the head of IT, vice chancellor, chief laboratory manager, etc.). Therefore, to maintain anonymity, demographic data for these interviewees is not presented. Interviews with consortia members (e.g. principal and co-principal investigators; PhD students) who were not available at the time of the site-visit, were conducted remotely via skype or phone calls.

### Data collection

In-depth semi-structured interviews conducted during site visits and via skype or phone calls, constituted the main source of data collection. The research team also carried out three focus group discussions with a total of 13 ACBI-affiliated PhD students. All data were collected between May 2015 and August 2016. Other sources of data such as field notes, institutional documents and reports were reviewed and used to provide background information and/or to verify information provided through the interviews and focus group discussions [27] (S3 Supplementary File: A list of the documents reviewed).

Prior to each site visit, the purpose and process of the research was explained to each of the principal investigators in the African research institutions. All 30 African principal investigators were invited to complete an on-line questionnaire to obtain information on their professional expertise and experiences, as to provide a broad overview of the current research capacity in their department or research institution. The questionnaire covered the following themes: demographic information; professional development; research and training capacity at institutional level; partnerships and collaborations; main strengths and gaps/challenges, and possible solutions (S4 Supplementary File: Pre-site visit online questionnaire). This information was also used to indicate areas for particular emphasis during the interviews and to supplement information obtained from on-site visits. Overall 27 out of 30 principle investigators responded to the online questionnaire. Logistical arrangements and interview schedules for each of the site visits were all planned in coordination with the principal investigators.

For the purpose of the in-depth interviews, five separate interview guides were developed; one for each of a) the principle investigators or co-principal investigators, b) the PhD supervisors, c) the PhD students, d) the Heads of Department/Institute Deans or Principles, and e) the other research and institute support staff (S5 Supplementary File: Compiled interview guides). In total, eight institutions were visited in six African countries. Two institutions were visited in Ghana, two in South Africa, and one in each of the Democratic Republic of the Congo (DRC)—Kinshasa, Botswana, Senegal and Zimbabwe. Within these eight institutions, nine different departments that hosted ten research consortia were assessed. One of the departments hosted two consortia under two different research groups. To validate findings and cross-check whether similar capacity gaps are found elsewhere, further data concerning African institutions that were not visited were obtained from the ACBI-affiliated principal or co-principal investigators and doctoral students during consortium-wide meetings within and outside the UK, or remotely by phone or Skype interviews.

Overall 86 in-depth interviews with unique interviewees and three focus group discussions involving 13 ACBI-affiliated PhD students, were conducted face-to-face during on-site visits to consortia and by remote communication. The majority of participants were principal investigators and PhD students; others included academics, laboratory staff, research and postgraduate managers and support staff such as accountants and laboratory technicians (Table 1).

**Table 1:** Types of participants in in-depth interviews.

Participant type	Number of participants across 10 consortia by award rounds 2015 & 2016	
	Award Holders’ Round 1–2015 (interviewees across 5 research consortia)	Award Holders’ Round 2–2016 (interviewees across 5 research consortia)
ACBI-affiliated Principal investigator	18	10
ACBI-affiliated PhD student	12	10
Academic Supervisor/Head of Department	6	2
Laboratory technician	6	1
Dean/Provost	3	1
Vice Chancellor	1	0
Director of Administrative Department	1	1
Representative from the Research Office	3	2
Representative from the Graduate/Doctoral School	4	2
Finance officer/ accountant	2	0
Human Resources’ officer	0	1
**Total**	**56**	**30**

Interview duration ranged between 30–90 minutes depending on the type of information required from each interviewee. Interviews were carried out until data saturation was achieved. Data from the interviews and FGDs informed qualitative assessments for eight institutions. The consortia to be funded had been selected during two annual rounds in 2015 and 2016. Five of the institutions that participated on our study were part of the Round One 2015 cohort and three institutions were part of the Round Two 2016 cohort. Table 1 below shows who was included in the in-depth interviews (excluding FGDs) across these institutions.

### Data quality assurance and analysis

To maximise the quality of data obtained, whenever possible each interview and focus group discussion was carried out by two researchers and information obtained was verified by at least two sources (e.g. other PhD students or academic staff in the same research institution) to enhance validity. Interviews and focus group discussions were audio recorded (if permission was given) and extensive notes were taken. These notes were written up within 24 hours of the interview and were compared between the researchers, and with the audio recordings, to verify accuracy. An independent advisor also reviewed the data collection tools and a sample of the reports.

All authors involved in this study have backgrounds in social sciences and public or global health and are well trained researchers experienced in qualitative research. Data collection was mainly led by Dr Stefanie Gregorius (SG) and Professor Imelda Bates (IB) with the support of CCR’s programme manager who was mostly observing and taking notes during the interviews. Data transcription, manual coding, and analysis was mainly led by SG and was discussed with other authors from the research team for verification (i.e. ensuring they align with the assessment criteria) and finalisation. Individual institutional reports were reviewed by key stakeholders involved in the study. Their comments and input were incorporated in each of the institutions’ final reports and were used as a source of data for this manuscript.

Data from interviews and FGDs were analysed using a framework approach [28] (S6 Supplementary File: Thematic framework used to analyse institutional capacity strengths and gaps). Analysis was conducted for each institution individually. Themes were identified in advance based on the benchmark which informed the semi-structured interview guide (S5 Supplementary File). The framework was based on the benchmark which was informed by good global practice for research systems. The findings from each visit were collated into a narrative institutional assessment report which highlighted the research capacity strengths, weaknesses, and described examples of innovative or good practice and problem-solving. Each institutional report also included suggestions from interviewees about how to address gaps in their institutional research capacity based on discussions between consortia stakeholders and the CCR research team during consortia meetings and workshops. The draft institutional reports were sent to the relevant principle or co-principal investigator to be shared with stakeholders interviewed for their review and input before they were finalised. These reports were provided confidentially to the consortium and the grant management team and all reports were anonymised before they were shared beyond these two groups. Findings from all institutional reports were combined into one narrative report.

### Ethical considerations

Ethical approval for this study was granted by the Research Ethics Committee of the Liverpool School of Tropical Medicine (Ref: 13.14RS) on condition that the head of each institution provided a support letter- as currently there is no national or international guidance about how to deal with ethical approval for these low risk, multi-country studies which are at the interface between evaluation and research. The lead researcher for each African institution, and their institutional heads consented in writing to participate in this study by signing ‘institutional approval forms’ which explained the aim of the research, the study procedure, and the risks, benefits and maintenance of confidentiality.

At the individual level, informed verbal consent was obtained from each interviewee after explaining to them the purpose of the study, the type of questions that would be asked, research procedure, voluntary participation, recording purposes, confidentiality and privacy, risks, benefits and dissemination of findings. Participants were free to withdraw at any time. The participants understood that although the findings would be helpful for informing capacity strengthening plans for their institutions, the ACBI programme remit did not include providing funding for them to implement solutions to research capacity gaps that were identified through the study.

## Results

This section presents the consolidated findings from all the institutional assessment studies by research capacity thematic areas. Information obtained from the interviews was mapped onto the framework under the six themes (see S6 Supplementary File) and details of strengths and gaps under each of these thematic areas were disaggregated. Full details of the strengths and gaps in research capacity that emerged from the data for each theme, as well as examples of good practices and problem-solving that may be useful to other institutions, are provided in S7 Supplementary File.

All participants were assured of anonymity. For the purpose of this manuscript, quotes have only been ascribed to ‘Principal investigators’, ‘PhD students’ and ‘others’, where ‘others’ refer to all other stakeholders (see Table 1). Institutions have been allocated identifiers indicating whether they were in the first round of awards (e.g. R1X) or the second round (e.g. R2X) where X is a unique letter code for each institution (see S7 Supplementary File).

### Research infrastructure and facilities

Study spaces for researchers and PhD students were generally considered to be adequate though the quality could be improved by separating postgraduates from undergraduates and by providing work space outside of laboratories. Traditional book libraries were rarely used by postgraduates and researchers as they preferred electronic resources despite the limited availability of up-to-date and subject-specific journals. Many participants considered that accessing the literature through consortia was a major benefit of being part of the ACBI but was not perceived as a sustainable solution to the lack of funding for journal subscriptions in African institutions. Although many institutions did have free or low-cost access to an extensive array of academic journals online (e.g. through ‘Research4Life’) this access tended to be under-utilised and there was a recognition that more efforts may be needed to alert researchers to this resource.

Several institutions had problems with unstable electricity supply which adversely affected research activities and many did not have backup generators or solar power. Whilst all institutions involved in the study had free Wi-Fi access for staff and students, the reliability and quality of the internet varied and there were frequent interruptions. This was particularly challenging for research groups working with large data sets and modelling and who therefore required a stable high-speed connection. Stable electricity supply and access to internet was variable and was a significant challenge for some students, resulting in serious delays in their research work, as indicated in the quote below.

*“… The internet is a big challenge*. *The Internet is not even meant for students*, *it is only meant for the faculty members*. *As a PhD student I do not have access*. *Students normally buy personal modems*. *I bought a modem that I use when I’m (here)*, *and without that I cannot access the internet… I do not see this is changing for now*. *…*. *Electricity is also a challenge*. *It is getting worse… At times I go to school*, *even at home it’s not good*. *Not that what I want to do cannot be done- I could have done it easily on my computer at the comfort of my room- but because of the light aspect I need to go to school… sometimes no electricity till the end of the day*. *At times it can be frustrating*.*”*

[PhD student]

In many institutions, institutional email services were dysfunctional, and staff used personal email addresses for work-related communications. Improving institutional email systems did not appear to be a priority for action or funding by institutions, although it emerged as a critical cross-cutting bottleneck in institutions’ ability to communicate effectively with staff.

Laboratories appeared to be one of the weakest and most neglected components of research systems in the institutions despite being essential for much natural sciences research. Inadequate laboratories had prevented some types of research from being conducted or had necessitated procurement of external laboratory services. In many institutions, the physical laboratory space was insufficient, equipment was lacking or poorly maintained, the supply chain for laboratory consumables was unreliable and the procurement process was cumbersome and slow.

*“…it’s so embarrassing*. *This was once a lab*. *And I now call it ‘city plastic’*. *We use empty plastic bottles as substitute for glassware*. *This is due to the failure to get money for equipment since a long time*. *We haven’t bought any new equipment in 10 years …”*

[other]

Institutions also reported challenges in procuring laboratory consumables and equipment. Inefficient procurement systems and/or complex custom regulations combined with a lack of institutional and national funding can cause significant delays to the research, particularly among postgraduate students. For example, some consumables and equipment were reported to be delayed by six months to two years. The following quote highlights the frustration with inefficient procurement systems:

*“We have to buy the chemicals from Europe very often and bring them into the country and pass through rigorous customers clearance procedures… it could take half a year to get the same chemical that you can get in an hour here [in the UK]*. *it’s very very frustrating especially for PhD students… It can take months to clear the chemicals or equipment from the airport to get them here…*. *This has serious precautions on research outputs also*. *The amount of time it will take for a student to do reasonable work is frustrating- so we’ve compared over the years what students can do when they’re in a European setting and what they can do when they’re back home… …We can only do the basic analysis work on the samples but not the sophisticated work*. *We send it to specialised laboratories abroad (e*.*g*. *in Sweden) …”*

[Principal investigator]

Laboratory management systems including quality assurance, standard operating procedures and health and safety were also generally of inconsistent or poor quality. Participants perceived these laboratory-related challenges as major barriers to the progress of their research. It affected their ability to produce high-quality research and, for some, their ambition to achieve international laboratory accreditation.

### Teaching and learning

All institutions offered training courses for postgraduate research students and in some institutions these were compulsory, though the quality was reported to vary depending on the course and institution. Formal training needs assessments, availability of courses and funding for training for academic staff, were generally limited or absent. Doctoral students commonly cited their training needs as research methods, data analysis software, geographic information systems and academic and proposal writing. Non-Anglophone PhD students also identified a need for training in English language to help them overcome language barriers. Such language barriers were demonstrated when students attended or presented their research work at international scientific conferences where English is the main language of communication; when accessing scientific literature; and when communicating or networking with other PhD students and consortia members. Some participants also stressed the need for gender equity at all stages of PhD programmes:

*“…I think there needs to be gender awareness training in science*. *Women have less support than men in research*. *I would like to build the capacity of women*. *I am female*, *but I can do a PhD*, *I can help others*. *I can be a role model for other females who want to do a PhD …”*

[PhD student]

Even when training was available, the high teaching and administrative workload of researchers often made it difficult for them to attend. Grant-funded students tended to receive more external training than self-funded students. However, there were several examples of how grant-funded students shared their resources (e.g. internet access, conference proceedings, collaboration networks) and new knowledge with less well-funded students who had limited opportunities. This type of sharing and cascading appeared to be a widespread phenomenon.

Some institutions had graduate schools responsible for organising postgraduate courses. However, there were generally limited opportunities and funding for training in generic research skills such as proposal writing, intellectual property issues, quality assurance and research ethics. Despite having research ethics committees in most institutions, knowledge among participants about ethical issues, policies and procedures related to ACBI natural sciences subjects was limited. Staff within research offices and graduate schools themselves also expressed their need for more financial resources to support their work and training in how to support research and researchers. Training opportunities for research support staff, and particularly for laboratory technicians, were generally neglected compared to opportunities for academic staff. It was recognised that lack of professional development for laboratory technicians, most of whom were responsible for both teaching and research laboratories, could negatively impact the progress and quality of the doctoral students’ research work.

### Research strategies and support

All institutions had medium- to long-term (3–10 year) research strategies that had been developed through wide consultation and which were aligned to national needs. However, research priorities at departmental level often more closely reflected the interests of senior researchers and were therefore not necessarily matched to national needs. Most participants acknowledged the importance of effective departmental strategic plans, but the development and implementation of these plans was hindered in some institutions due to a lack of expertise in strategic planning. Some participants suggested that students and non-academic staff (e.g. laboratory technicians, support staff) could be more involved in strategic planning activities to ensure that their needs were met. Others suggested that consortium partners with effective departmental strategic plans could support less experienced partners through sharing of resources or examples of good practice such as carrying out departmental reviews to improve activities in the department:

*“… our departmental review*, *I think it’s every five years*, *they appoint external reviewers to review our department*. *So*, *the reviewers interview the Head of Department*, *academic staff*, *some postdocs*, *some postgrads*, *some undergraduate students*, *and they then compile a report and we read the report and we look at what the recommendations are of the report and then we try and improve on those things or implement those recommendations*.*”*

[Principal investigator]

There was almost no use of electronic tracking systems to generate institutional level data about research activities. Most institutions had research offices or units to help researchers at the pre-, intra- and post-award stages of the research process. However, their roles and structures varied across different institutions and the services they provided were often not well communicated. This meant that researchers were not clear about how to make best use of this support.

### PhD programmes

The application and admission processes for doctoral courses was generally organised through Graduate Schools and the application process was similar across institutions. However, the registration process varied widely and some institutions required an upgrade from MPhil to PhD registration. In some cases, the initial registration process could take up to one year and was poorly understood partly due to poor communication, misunderstandings and lack of guidelines. PhD handbooks were generally available and of reasonable or good quality, but students were often not aware of their existence. All institutions offered inductions for new students (generally twice per academic year) but they were mainly targeted at undergraduate students and there was a lack of postgraduate-specific inductions:

*“…The University doesn’t have so much postgraduate students*, *so there is a lack of services that support them*. *So*, *what happens is*, *one of the issues is*, *like*, *open day*, *we don’t have an open day or orientation specifically for postgrads*. *But it would be really useful to know what the guidelines and expectations are …”*

[PhD student]

Most institutions had guidelines for PhD supervisors and there was awareness of the need for good quality supervision of research, although this was sometimes compromised by supervisors’ heavy teaching and administrative loads. Over the last few years, most institutions had increased the number of staff with PhDs and had provided training for research supervisors. Institutions were increasingly requiring PhD students to publish papers prior to their final examination. Overall the relationship between the PhD students and their supervisors, and the quality of supervision, was considered satisfactory. There was variable understanding of the need for, and role of, mentors, but also some clear examples of where mentors were perceived as advantageous, such as for students who did not feel comfortable discussing financial or gender-related issues with their supervisor.

All institutions monitored students’ progress either through submission of annual progress reports, or through students presenting their work at regular departmental seminars and workshops. Participants recognised that the ACBI funds had accelerated their progress compared to self-funded students who often had to take on extra teaching duties to supplement their income. Students noted that they could encounter long delays in the final PhD exam mostly due to lack of timely feedback from external examiners, and universities are beginning to address this by offering, or increasing the value of, honoraria or by focusing efforts on the more efficient examiners.

### Financial management and funding

Most institutions had clear guidelines for financial management of research though responsibility for managing project finances varied. Most ACBI PhD students were not aware of the level of funds that were available for their research, there was variation in the monthly stipend they received, and there was generally a lack of institutional guidance about stipend rates or PhD students. In a few institutions, participants had experienced challenges in managing large international grants and in a small number of cases, African principal investigators preferred funds to be handled by the UK institution. On the other hand, there were several examples of delays in transfer of funds from the UK to African institutions. Reasons for these delays included limited experience among some UK institutions of working with this kind of funding schemes and problems with disbursement of funds within the African institutions. A few participants suggested that capacity strengthening activities to better manage international funding schemes should also target UK institutions. For instance, some suggested that UK principle investigators should share relevant experiences with each other at the Award Holders’ Meetings. These cash flow issues were common and had a significant negative impact on research activities. In the African institutions external grants were typically managed in a project-specific account and updates were generally not provided automatically or regularly to principal investigators. Both UK and African principal investigators were responsible for tracking their spend against budget and most did not receive administrative support for financial management of their projects. Institutional overheads on grants were generally in the range of 2–10% but there was lack of clarity and understanding about how these were used or distributed across the institution and departments.

Inefficient institutional procurement systems were a significant factor in delaying research activities, especially in relation to laboratory consumables:

“*The finance department is unpredictable*. *The last time we received money for equipment was to facilitate the commencement of MSc programmes*. *Getting all the required quotations and feedback was a nightmare*. *And the time given to process the purchases wasn’t reasonable and the money was forfeited*.*”*

[other]

### Research collaborations

In most cases, African principal investigators felt that they had been equitably involved in generating research questions and priorities for the application to the ACBI programme. They highlighted respect, complementary skills, effective communication and strong leadership as important for their successful research partnerships. All participants recognised that collaborations, including North-South, South-South, Anglo-Franco and within-institutions’ departments and faculties, were important for strengthening research capacity, but that there was room for improvement:

*“…our offices are next to each other in one corridor*, *but I don’t know much about what research exactly my colleagues are doing in our department*. *There should be more communication*. *We should come together more and work together*…”

[Principal investigator]

International staff exchanges and participation in international conferences were recognised as particularly important for increasing visibility and promoting research collaborations and partnerships. Some institutions reported having dedicated offices and staff to promote and increase international partnerships and links with industry. Laboratory collaborations with public and private sectors were noted as valuable for promoting institutional research capability and there were particularly good examples of government-funded laboratories in research institutions that were very experienced and had strong links with many stakeholders.

### Suggestions and examples of how to improve institutional research capacity

Participants offered many practical suggestions for ways to improve the research capacity within their departments and institutions. These are detailed in S7 Supplementary File and those that are particularly transferable to other institutions are summarised in Box 1.

**Box 1. Practical suggestions for ways to improve institutional research capacity.**

- Formalise PhD induction processes, possibly including peer-to-peer interactions involving more senior PhD students, and ensure post-graduates and under-graduates have separate study spaces
- Establish formalised, mandatory courses for PhD students including research methodology, critical thinking and appraisal skills, ethics and gender awareness/diversity training
- Maximise training opportunities by opening up project-specific course, workshops etc to non-project staff
- Establish units to help researchers find funding opportunities and to critically review proposals
- Provide regular financial reports for principal investigators on project budgets; improve within-institution standardisation and clarity on use of project overheads and PhD stipends
- Include funds for employing and training administrative, finance and laboratory research support staff in grant proposals and encourage sharing of skills and experiences of these staff beyond the project and department
- Promote strategies to increase South- South collaborations especially across language barriers
- Consider establishing Doctoral Schools with a remit to drive strategic research areas and strengthen national and international research collaborations
- Establish effective institutional email system, which will facilitate institutional communication on all issues including research
- Separate the teaching and research laboratories; plan equipment purchases and maintenance strategically and for some institutions, consider investing in achieving laboratory ISO accreditation to improve research quality and as a potential source of revenue
- Improve alignment of national needs and institutional research activities through improved efforts at communication between those generating and those using research

During the interviews, interviewees were prompted to provide examples of good or innovative practice in strengthening their research capacity, and solutions for solving some of their research capacity challenges. Details of these are provided in S7 Supplementary File and selected examples from across all six themes which may be particularly useful for other institutions, are presented in Box 2.

**Box 2. Examples from participants of good or innovative practice and problem-solving to strengthen research capacity.**

- Participatory strategic planning meetings/workshops, involving academic and support staff (e.g. managers, technicians) covering SWOT analyses and implementation plans with clear performance indicators and pre-defined targets
- An institutional research committee to periodically review research themes to ensure they meet local needs
- Invitations to policy makers, industry, NGO other research institutions to workshops to ensure research is relevant to local needs
- Regular external departmental reviews to promote implementation of activities to improve the department
- Institutional internationalisation policy and specific offices/departments in place to increase international partnerships
- Development of departmental newsletters about current research shared within and across departments
- Student-supervisor contracts that include clear roles and responsibilities, targets and objectives with times lines, training schedules and meeting dates
- Investment in strengthening research quality assurance systems with dedicated quality assurance units and the development of quality assurance documents
- Revenue from selling laboratory manuals to undergraduate students is reinvested in supporting training for PGR students
- Peer-to-peer progress monitoring system for PhD students, including regular Skype calls and WhatsApp groups, to check on each other’s progress
- Mechanisms to encourage cascading of opportunities and information from funded to non-funded research students
- Development of laboratory inventories across all partner institutions to make smart decisions about purchase and utilisation of equipment

## Discussion

This study describes an assessment of the research capacity of African institutions against a benchmark, using information from multiple perspectives within the institutions (i.e. from senior officials and academics to research students, laboratory technicians and research support staff). The assessment covered all the components that need to be in place for an institution to undertake international-standard scientific research. The use of the same benchmark, research methods and data collection tools across the different institutions not only assisted the institutions themselves to gain a holistic view of their research capacity, but it also enabled commonalities in research capacity strengths and gaps among the institutions to be identified. These could be useful targets for external development agencies.

Despite the importance of understanding the landscape of existing capacity for conducting high quality natural sciences research in Africa, we were unable to find any published studies that had systematically documented institutional research capacity for natural sciences in Africa. The few published studies on assessments of research capacity in African research institutions have focused on health research [23, 24, 29] or on quality assurance systems [30–31]. Gaps in research capacity in African institutions have been described in the literature, predominantly in relation to specific areas of health research. These studies covered health policy and system research [29, 30, 32], public health research [33], health research management and support systems [23] and doctoral programmes [24]. Although they focussed on health research, the gaps in research capacity described in these papers were similar to those that were identified through our study and ranged from a lack of policies and strategies, limited training opportunities and problems with space, resources and connectivity, to weak laboratories, and cumbersome and slow procurement processes.

A lack of study, office and teaching spaces [24, 29], irregularities in payments to PhD students [23], limited access to research resources such as books, journals and articles [24] and insufficient personal computers, computer software, and access to effective internet [24, 29, 30] have been described in African universities in the context of doctoral training programmes and health policy research. Functional, well-equipped laboratories are key to conducting experiments and carrying out quality research. Inadequate laboratory capacity to support research, such as lack of equipment, poor maintenance and inadequate space have been described in other studies in relation to PhD programmes and science and technology research [23, 34, 35]. Inefficient procurement systems coupled with complex customs regulations appear to be common time-consuming challenges that result in significant delays and are a major hindrance to the efficient management of research projects [23]. Achieving international laboratory accreditation is an aspiration for some institutions since it would improve their competitiveness and help to attract international research partners. A stepwise process to achieve accreditation, as has been done for African diagnostic laboratories [36] and blood transfusion services [37], may be an option. Our study found that professional development of laboratory staff is often overlooked. As laboratory staff are key members of many research teams it is important to find ways to empower them and to develop their skills to maintain laboratory equipment and manage quality systems. Opportunities for sharing equipment and for developing laboratory technicians emerged from our study as one of the most important benefits of belonging to a multi-national research partnership such as the ACBI.

The limited research-related training opportunities in Africa and lack of coordinated institutional training for researchers, academic and non-academic staff, including laboratory technicians [23] and PhD students [24] that we found in our study have been described in other studies. The high teaching and administrative workload experienced by research staff can impact negatively on the quality of their own research and their ability to supervise the research of others including PhD students [23]. Our findings, and those from other studies, indicate a need for regular appraisals and formal reviews of individuals’ training needs. This would help to streamline and institutionalise high-quality research-related training and make sure it is tailored to the needs of the research staff.

Our study, and a previous study, found that PhD students experienced slow registration processes, inadequate or absent inductions, and delays in completing final PhD examinations [24]. We found evidence that some institutions are beginning to address the latter through increased incentives for external examiners. At institutional level, and similar to previous reports, we found little evidence that research priorities at department level were aligned to national needs. There was a general lack of research strategies and no electronic tracking of research activities within institutions [23]. A consistent and very positive finding from our study, which has been reflected in previous reports [29,38], was the benefit expressed by participants of belonging to a multi-partner research consortium, and to a larger programme. In particular, the nature of the ACBI, with annual programme-wide meetings, and regular consortium level meetings and communication platforms, fostered both North-South and South-South partnerships which facilitated joint publications and funding applications. Further research comparing the effectiveness of research consortia compared to other models for strengthening research capacity strengthening would be helpful in justifying expansion, or not, of investment in research consortia.

Our assessment study had several strengths. The research process took account of the ACBI’s theory of change (S2 Supplementary File) to make sure that the topics covered during data collection were aligned with the overall strategy of the programme. The use of a benchmark and a single set of data collection tools meant that comparisons could be made across institutions allowing common strengths and gaps to be identified. Information was gathered from a range of cadres of staff involved in research in the institutions, so the data reflects multiple perspectives. The information provided by participants was verified by asking the same questions of more than one person which gives confidence in the validity of the findings. The assessments were designed to provide high-level information so the study did not generate in-depth information on any particular theme. However, where appropriate, we provided institutions with information about more detailed processes for in-depth assessments (e.g. for stepwise laboratory accreditation, good financial management, PhD training). The institutions were selected for on-site visits opportunistically based on the timing and location of consortium workshops in order to maximise interactions with all members of each consortium. This meant that some institutions did not receive a visit and some stakeholders were unavailable or had limited time which may have resulted in some missing information from institutions. Findings from this study have already resulted in several improvements to the ACBI programme including more flexibility in the use of funds, refinements to the application form, increased emphasis on enhancing laboratory capacity and the establishment of a programme-wide communication platform.

In common with a previous study, our study found that the majority of gaps in research capacity within institutions can be addressed at little or no cost by, for example, providing in-house training, establishing research seminars or developing policies, guidelines and handbooks [23]. The widespread but hidden phenomenon of cascading of information and resources from well-funded to less well-funded students would be worth exploring in more depth to understand how this could be utilised to widen participation and improve equity among students. Some of the more intractable challenges in strengthening their research capacity facing institutions in Africa will take time to address and will need external funds. However, there are relatively few funders and international organisations that focus on system-level research capacity strengthening [39]. These agencies have called for a “significant re-think of the approach to capacity development” and a shift from reliance on external funding towards more national support from African governments with more southern-led agendas and research management [40–41].

There is understandable caution about investing significantly in strengthening research capacity without standards and metrics against which such investments can be measured. Our study has identified specific areas that the institutions themselves find particularly challenging and which are recurring issues across diverse institutions involved in natural sciences research which could be targets for investment (Box 3). These areas are very similar to those that emerged from previous capacity assessments for health research and, as has been done in a previous programme [23], indicators could be developed for these areas that would enable progress to be tracked if funds were available to remedy the capacity gaps.

**Box 3. Research capacity areas for advocacy and/or funding that need external support and which are common needs among institutions undertaking natural sciences research.**

- Sufficient, high-quality space for study, teaching and laboratory work
- Access to efficient internet services, computers and electronic resources for research
- Access to appropriate, well-maintained laboratory equipment and reliable and timely procurement systems
- Training for researchers based on needs assessments which include ‘soft’ skills (e.g. ethics, critical thinking, leadership)
- Professional development for research support staff including laboratory technicians, which is commensurate with the investment in researchers
- Alignment across regional/national research priorities, research funding and research generation to facilitate utilisation of research findings
- Consolidation and expansion of opportunities for South-South and South-North international research collaborations
- An agreed standard and accreditation programme for institutional research management systems

The fact that many institutions face similar challenges adds strength to the argument for joint external investment in generic weaknesses in research capacity. Investing in remedying weak areas in research systems across many institutions is likely to provide better value-for-money than providing fragmented, non-strategic support for individual institutions or departments, and plays to the unique strengths of alliances of development funders. An example of how funders, policy makers and researchers can jointly support improvements in research systems is the innovative new Good Financial Grant Practice programme designed to strengthen Africa’s research and development infrastructure [42]. This programme has developed a best practice standard for the financial governance of grant funds awarded to grantees to standardise, simplify and strengthen the governance of grant funding [42]. Suggestions for a similar programme in research management systems [43, 44], with staged levels to achieve a standard, are at an early stage, but would complement this financial programme and also help to inform funders’ and governments’ investments. There is a paucity of high quality studies to underpin such a standard and to guide strategic and measurable activities to strengthen research capacity in African institutions. Since the research capacity gaps are similar across many different institutions, efforts should now focus on gaining an in-depth understanding of how to overcome these challenges while avoiding any unnecessary duplication of effort. This research should be robust and carefully designed, using standard benchmarks and prospective data collection to improve science and technology research capacity and ultimately to impact on the socio-economic development in sub-Saharan Africa.

## Conclusion

Improving capacity for research in natural sciences in low-and-middle-income countries is essential to drive socioeconomic development and to achieve the Sustainable Development Goals. The innovative use of a benchmark and standardised tools in our study highlighted gaps in institutional research capacity that are particularly challenging to remedy and which are common to many institutions. These gaps, such as neglected research laboratories, little opportunity for creating collaborations and networks, and poor-quality facilities, are generic since they are similar irrespective of the research focus of the institution. These gaps could therefore be effective targets for national governments and international organisations that invest in strengthening research capacity in Africa since such investments will have wider benefits beyond the scope of the programme.

## Supporting information

S1 Supplementary File List of African countries where ACBI is implemented. (DOCX)

S2 Supplementary File Theory of Change diagram for ACBI. (DOCX)

S3 Supplementary File List of documents reviewed. (DOCX)

S4 Supplementary File Pre-visit online questionnaire. (PDF)

S5 Supplementary File Interview guides. (PDF)

S6 Supplementary File Thematic framework used to analyse research capacity strengths and gaps. (DOCX)

S7 Supplementary File Strengths and gaps in research capacity that emerged from the data for each theme, with examples of good practices and ways of solving problems that may be useful to other institutions. (DOCX)
