# Supplementary material for: Strengthening capacity for natural sciences research: A qualitative assessment to identify good practices, capacity gaps and investment priorities in African research institutions
Source: PLoS One. 2020 Jan 24;15(1):e0228261. doi: 10.1371/journal.pone.0228261 (PMC6980527; doi:10.1371/journal.pone.0228261)
Supplement: S1 Supplementary File — (DOCX) [file pone.0228261.s001.docx]

**S1_Supplementary file 1:** **List of African countries where ACBI is implemented**

| **#** | **Country** | **#** | **Institutions** | **#** | **Department** |
| --- | --- | --- | --- | --- | --- |
| 1 | Botswana | 1 | University of Botswana | 1 | Chemistry Dept. |
| 2 | Burkina Faso | 2 | Institut de l'Environnement et des Recherches Agricoles (INERA Research Institute) | 2 | Dept. Ecology Management and Natural Resources |
| 3 | Cameroon | 3 | University of Yaoundé I | 3 | Chemistry Dept. |
|  |  |  |  | 4 | Dept. of Inorganic Chemistry |
| 4 | Congo | 4 | Université Marien Ngouabi, Faculté des Sciences et Techniques | 5 | Université Marien Ngouabi, Faculté des Sciences et Techniques |
| 5 | Democratic Republic of the Congo-DRC | 5 | University of Kinshasa | 6 | Dept. of Natural Resources Management |
| 6 | Gabon | 6 | Gabonese National Parks Agency | 7 | Gabonese National Parks Agency |
| 7 | Ghana | 7 | KNUST | 8 | Dept. of Computational Chemistry |
|  |  |  |  | 9 | Dept. of Crop & Soil Sciences |
|  |  |  |  | 10 | Dept. of Chemistry |
|  |  | 8 | FORIG | 11 | FORIG |
| 8 | Kenya | 9 | University of Nairobi | 12 | Dept. of Geology (Inst. Climatic Change and Adaptation) |
|  |  | 10 | Maseno University | 13 | Dept. of Chemistry |
| 9 | Malawi | 11 | Lilongwe University of Agriculture and Natural Resources (LUANAR) | 14 | Lunyangwa Agricultural Research Station |
| 10 | Mauritius | 12 | University of Mauritius | 15 | Mechanical and Production Engineering Dept. |
| 11 | Namibia | 13 | University of Namibia | 16 | Dep. of Chemistry and Biochemistry |
| 12 | Nigeria | 14 | Federal University of Agriculture Abeokuta (FUNAAB) | 17 | Dept. of Soil Science and Land Management |
|  |  | 15 | University of Lagos | 18 | Dept. of Mechanical Engineering |
| 13 | Senegal | 16 | UCAD- Cheikh Anta Diop University | 19 | School of Water and Water Quality |
| 14 | South Africa | 17 | University of Zululand | 20 | Chemistry Dept. |
|  |  | 18 | Rhodes University | 21 | Institute for Water Research |
|  |  | 19 | University of Western Cape; Energy Materials, Materials Science and Manufacturing | 22 | CSIR- Council for Scientific and Industrial Research |
|  |  | 20 | University of Pretoria | 23 | Dept. of Mechanical and Aeronautical Engineering |
|  |  |  |  | 24 | Department of Zoology and Entomology |
| 15 | Tanzania | 21 | University of Dar Es Salaam | 25 | Dept. of Water Resources Engineering |
|  |  | 22 | Dar es Salaam Institute of Technology | 26 | Dar es Salaam Institute of Technology |
| 16 | Uganda | 23 | Makerere University | 27 | Dept. of Geology and Petroleum Studies |
|  |  | 24 | Centre for Research in Energy and Energy Conservation | 28 | Centre for Research in Energy and Energy Conservation |
| 17 | Zambia | 25 | University of Zambia | 29 | Zambia Agriculture Research Institute (ZARI) |
| 18 | Zimbabwe | 26 | University of Zimbabwe | 30 | Dept. of Soil Science and Agricultural Engineering |
| **Total** | **18 countries** | **Total** | **26 Institutions** | **Total** | **30 Faculties/dept/centres** |
