# Supplementary figures and images for: Strengthening capacity for natural sciences research: A qualitative assessment to identify good practices, capacity gaps and investment priorities in African research institutions
Source: PLoS One. 2020 Jan 24;15(1):e0228261. doi: 10.1371/journal.pone.0228261 (PMC6980527; doi:10.1371/journal.pone.0228261)

**S2_ Supplementary file 2: Theory of Change diagram for ACBI**

**
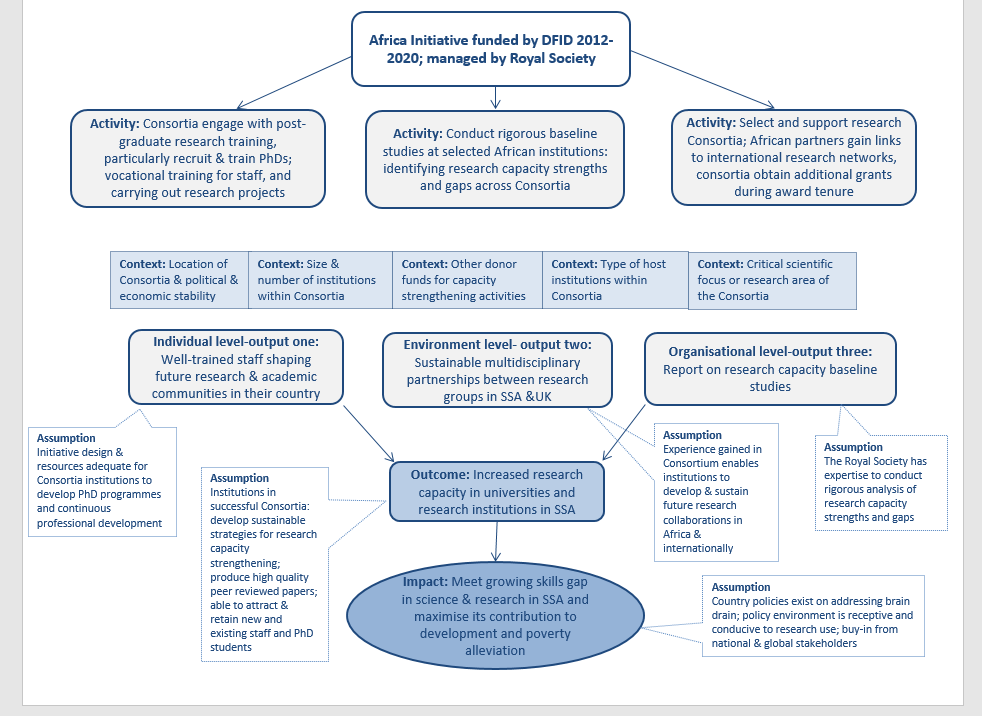
**

Supplement: S2 Supplementary File — (DOCX) [file pone.0228261.s002.docx]
