# Supplementary material for: Strengthening capacity for natural sciences research: A qualitative assessment to identify good practices, capacity gaps and investment priorities in African research institutions
Source: PLoS One. 2020 Jan 24;15(1):e0228261. doi: 10.1371/journal.pone.0228261 (PMC6980527; doi:10.1371/journal.pone.0228261)
Supplement: S3 Supplementary File — (DOCX) [file pone.0228261.s003.docx]

**S3_ Supplementary file 3: List of documents reviewed**

**University A**

- University A - Intensifying Research Strategy – Annual Research Report 2008
- University A – University research Strategy
- University A – School of Graduate Studies Academic Calendar 2015/2016
- University A – Policy on Internationalisation
- Faculty of Science, Department of Chemistry Operational Plan 2016

**Institute B**

- Institute B Annual Report 2012 & 2013. Available [link to Institute B’s annual report]
- School of Graduate Studies, Entry Requirements and General Regulations for Graduate Programmes.
- School of Graduate Studies, Manual for Thesis Preparation for Award of Master and Doctoral Degrees, Dated November
- University B, Students Guide, June 2013.
- School of Graduate Studies, Progress Report on Postgraduate Diploma, Masters and Doctoral Degree Candidates

**University C**

- No documents reviewed

**University D**

- School of Graduate Studies, Entry Requirements and General Regulations for Graduate Programmes.
- School of Graduate Studies, Manual for Thesis Preparation for Award of Master and Doctoral Degrees, Dated November 2013.
- University D, Students Guide, June 2013.
- College of Agriculture and Natural Resources (CANR), College Strategic Plan 2005-2014, Dated April 2006.
- University D, Quality Assurance Bulleting, Volume 11, 2014.
- University D, Criteria for Appointments and Promotions of Senior Members (Academic).
- School of Graduate Studies, Progress Report on Postgraduate Diploma, Masters, and Doctoral Degree Candidates.
- School of Graduate Studies- copy of initial and follow-up letters issued to external examiners.

**University E**

- School of Graduate Studies, Entry Requirements and General Regulations for Graduate Programmes.
- School of Graduate Studies, Manual for Thesis Preparation for Award of Master and Doctoral Degrees, Dated November 2013.
- University E, Students Guide, June 2013.
- University E, Quality Assurance Bulleting, Volume 11, 2014.
- University E, Criteria for Appointments and Promotions of Senior Members (Academic).
- School of Graduate Studies, Progress Report on Postgraduate Diploma, Masters, and Doctoral Degree Candidates.
- School of Graduate Studies- copy of initial and follow-up letters issued to external examiners.

**University F**

- Royal Society – DFID application form (University F)
- University F Strategic Plan 2011-2015
- University F Quality Assurance Documents – [link to QA documents on University F website]
- Quality Assurance Laboratories
- Quality Assurance PostGrad
- Teaching and Learning Quality Assurance

**University G**

- Royal Society – DFID application form (University G)
- Capacity Research Unit - Research management support systems: review of capacity in MCDC’s partner institutions (RMSS) - Faculty of Medicine, Pharmacy and Odonto-Stomatology, University G, Location G, October 2014

**University H**

- RS/DFID research proposal
- CRU pre-visit questionnaire (completed by Location H PI)
- Project Title H: Inception workshop report
