# Supplementary material for: Strengthening capacity for natural sciences research: A qualitative assessment to identify good practices, capacity gaps and investment priorities in African research institutions
Source: PLoS One. 2020 Jan 24;15(1):e0228261. doi: 10.1371/journal.pone.0228261 (PMC6980527; doi:10.1371/journal.pone.0228261)
Supplement: S4 Supplementary File — (PDF) [file pone.0228261.s004.pdf]

Research Capacity Strengthening Questionnaire 2016 (Royal Society - DFID Africa Capacity Building Initiative)

Page 1: Welcome to the Capacity Research Unit's research capacity questionnaire 2016 for the Royal Society - DFID Africa Capacity Building Initiative

Dear participant,

The purpose of this survey is to collect baseline information about African partner institutions within the Initiative. This information will be followed up with individual interviews and is part of the Capacity Research Unit's research assignment to determine capacity strengths and gaps in postgraduate training and research.

For further questions please contact Dr Stefanie Gregorius (stefanie.gregorius@lstmed.ac.uk)

Many thanks for your collaboration!

Capacity Research Unit, Liverpool School of Tropical Medicine

Page 2: DEMOGRAPHIC INFORMATION

1. Was your consortium previously awarded a RS-DFID Network Grant? ✱ *Required*

- ○ Yes
- ○ No

2. What is the main scientific area to which your research belongs? ✱ *Required*

- ☐ Water and Sanitation
- ☐ Renewable Energy
- ☐ Soil-related research
- ☐ Other

2.a. If you selected Other, please specify:

3. Are you ✱ *Required*

- ○ Female
- ○ Male

4. How old are you? ✱ *Required*

- Under 25
- 26-35
- 36-45
- 46-55
- 56-65
- Over 65

5. What is your first language? ✱ *Required*

- English
- French
- Portuguese
- Swahili
- Other

5.a. If you selected Other, please specify:

6. Can you speak any other languages fluently?

- English
- French
- Portuguese
- Swahili
- Other

6.a. If you selected Other, please specify:

7. What country do you live in? ✱ *Required*

8. What is the highest level of academic qualification you have obtained? ✱ *Required*

- Certificate/ Diploma
- Bachelors
- Masters
- Doctorate
- Other

8.a. If you selected Other, please specify:

9. If you have a doctorate, how many years ago were you awarded it? ✱ *Required*

- 1-4 years
- 5-9 years
- > 10 years

10. How many years of research experience do you have post-PhD? ✱ Required

- 1-4 years
- 5-9 years
- > 10 years
- none

11. How many years of research experience do you have in total? ✱ Required

- 1-4 years
- 5-9 years
- > 10 years
- none

12. Which of the following best describes your primary place of work? ✱ Required

- Government department
- Independent Consultant
- Private business
- Research institution
- University/educational institution
- Other

12.a. If you selected Other, please specify:

13. Which of the following describe your major work role(s)? ✱ *Required*

Please select at least 1 answer(s).

- ☐ Consultant
- ☐ Director
- ☐ Government employee
- ☐ Lecturing/teaching
- ☐ Manager
- ☐ Research
- ☐ Other

13.a. If you selected Other, please specify:

14. Do you have any other professional responsibilities other than at your primary work place?

- ○ Yes
- ○ No

14.a. If yes, please specify

15. What percentage of your workload is devoted to research? ✱ *Required*

- 0%
- 1-33%
- 34-66%
- 67-100%

16. Please tell us about any staff, besides yourself as principal investigator, who are involved in activities funded by the award? (please split numbers by sex)

16.a. number of lab technicians (male/female) ✱ *Required*

16.b. number of PhD students (male/female) ✱ *Required*

16.c. number of post-doctoral researchers (male/female) ✱ *Required*

16.d. number of research assistants (male/female) ✱ *Required*

16.e. number of administrative staff (male/female) ✱ *Required*

16.f. number of finance officers (male/female) ✱ Required

16.g. Others, please specify

Page 3: PROFESSIONAL DEVELOPMENT

17. Please indicate the number of articles in international peer-reviewed journals you have contributed to through authorship in the last 10 years. ✱ *Required*

- 0
- 1-3
- 4-6
- 7-9
- 10-12
- > 12
- Other

17.a. If you selected Other, please specify:

18. In how many of these publications were you lead or last author? ✱ *Required*

- 0
- 1-3
- 4-6
- 7-9
- 10-12
- > 12
- Other

18.a. If you selected Other, please specify:

19. Please indicate the number of patents you have filed in the last 10 years. ✱ *Required*

- 0
- 1-2
- 3-4
- 5-6
- > 6
- Other

19.a. If you selected Other, please specify:

20. Please indicate the number of research grants you have been awarded in the last 10 years. ✱ *Required*

- 0
- 1-3
- 4-6
- 7-9
- 10-12
- > 12
- Other

20.a. If you selected Other, please specify:

20.b. How many of these other grants have been awarded in collaboration with other research institutes?

- 0
- 1-3
- 4-6
- 7-9
- 10-12
- > 12
- Other

20.b.i. If you selected Other, please specify:

20.b.i.a. How many of these grants have been awarded in collaboration with international partners?

- 0
- 1-3
- 4-6
- 7-9
- 10-12
- > 12
- Other

20.b.i.a.i. If you selected Other, please specify:

20.b.ii. How many of these grants were greater than $500,000?

- 0
- 1-3
- 4-6
- 7-9
- 10-12
- >12
- Other

20.b.ii.a. If you selected Other, please specify:

21. How many conference presentations have you delivered in the last 10 years? ✱ *Required*

- 0
- 1-3
- 4-6
- 7-9
- 10-12
- >12

- Other

21.a. If you selected Other, please specify:

21.b. Have you attended any professional development activities in the last 12 months? ✱
Required

- Yes
- No

21.b.i. If yes, how many?

21.b.ii. Please provide details of each event (title of event, where held, duration).

22. How are professional development activities currently funded at your institution? ✱
Required

- Institutional funds
- National competitive funding
- International funding
- Other

22.a. If you selected Other, please specify:

23. To what extent do you agree with the following statement: *I have had access to sufficient research training through my institution* ✱ Required

- Strongly Agree
- Agree
- Neutral
- Disagree
- Strongly Disagree

24. To what extent do you agree with the following statement: *I have sufficient skills to deliver research training at my institution.* ✱ Required

- Strongly Agree
- Agree
- Neutral
- Disagree
- Strongly Disagree

25. To what extent do you agree with the following statement: *I have sufficient resources to deliver research training at my institution.* ✱ Required

- Strongly Agree
- Agree
- Neutral
- Disagree
- Strongly Disagree

Page 4: RESEARCH AND RESEARCH TRAINING CAPACITY AT INSTITUTIONAL LEVEL

26. Does your faculty currently teach students? ✱ *Required*

- ☐ Undergraduate
- ☐ Masters
- ☐ PhD
- ☐ Other Postgraduate

26.a. How are these students funded? ✱ *Required*

- ☐ Self - funding
- ☐ Institutional funds
- ☐ Sponsorship
- ☐ Grant funded

27. How many of each student category have been taught and how many degrees awarded in the last 10 years?

[ ]

27.a. Undergraduate ✱ *Required*

[ ]

27.b. Masters ✱ *Required*

[ ]

27.c. Other Postgraduate ✱ *Required*

28. How many PhD students have you supervised in total? ✱ *Required*

29. How many of these PhD students have completed successfully? ✱ *Required*

30. How many PhD students are you currently supervising? ✱ *Required*

31. What research training is currently offered through your institution? Please select all that apply. ✱ *Required*

- [ ] Qualitative research
- [ ] Quantitative research
- [ ] Evaluation
- [ ] Grant writing
- [ ] Academic writing/publishing
- [ ] Conducting literature reviews
- [ ] Knowledge dissemination

- [ ] Knowledge translation (i.e., research into policy)
- [ ] Other

31.a. If you selected Other, please specify:

32. Does your institute/department have a strategic plan for research in place? ✱ *Required*

- [ ] Institutional
- [ ] Departmental
- [ ] Neither
- [ ] Other

32.a. If you selected Other, please specify:

33. If relevant, does your institute/department have a research ethics committee?

- [ ] Institutional
- [ ] Departmental
- [ ] Neither
- [ ] Other

33.a. If you selected Other, please specify:

34. Do you have access to research development funds at your institution/department? *
Required

- Institutional
- Departmental
- Neither
- Other

34.a. If you selected Other, please specify:

35. To what extent do you agree with the following statement: *I have sufficient administrative and financial support to complete my role as a researcher effectively.* *
Required

- Strongly Agree
- Agree
- Neutral
- Disagree
- Strongly disagree

Page 5: PARTNERSHIP/COLLABORATION EXPERIENCE

36. Prior to this Award have you collaborated with any of these (Programme Grant) partners before? (excluding Network Grants) ✱ *Required*

- Yes, all three
- Yes, 2 of three
- Yes, 1 of three
- No, none
- Other

36.a. If you selected Other, please specify:

37. Prior to this Award have you worked as part of a research consortium before? ✱ *Required*

- Yes
- No

38. Please state your level of agreement with the following statement: *All partners were equally involved in generating research questions and priorities for this Award Application.* ✱ *Required*

- Strongly Agree
- Agree
- Neutral

- Disagree
- Strongly Disagree

39. Please state your level of agreement with the following statement: *I fully understand the aims and objectives of our consortium and the research methods we will use.* ✱ *Required*

- Strongly Agree
- Agree
- Neutral
- Disagree
- Strongly Disagree

40. Please state your level of agreement with the following statement: *Within my research consortium my roles and responsibilities are clearly documented and understood by myself and all other consortium members.* ✱ *Required*

- Strongly Agree
- Agree
- Neutral
- Disagree
- Strongly Disagree

41. When was the last time the research consortium communicated as a group by telephone or Skype? ✱ *Required*

- In the last week
- In the last month

- Within the last 6 months
- Never

42. When was the last time the research consortium communicated as a group by e-mail? * *Required*

- In the last week
- In the last month
- Within the last 6 months
- Never

43. When was the last time your research consortium met face to face? * *Required*

- Within the last 3 months
- Within the last 6 months
- Less than 1 year ago
- More than 1 year ago
- Never

44. What do you see as your institution's three main strengths in conducting research? * *Required*

45. What do you see as your institution's three main weaknesses in conducting research? ✱ *Required*

46. How do you think these weaknesses can be improved? ✱ *Required*

Page 6: Thank you very much for taking part in this survey!

If you want to find out more about the work of the Capacity Research Unit (CRU) at the Liverpool School of Tropical Medicne, please visit http://www.lstmliverpool.ac.uk/research/cross-cutting-themes/capacity-research-unit/

If you have any questions please contact Dr Stefanie Gregorius (stefanie.gregorius@lstmed.ac.uk)
