# Supplementary material for: Strengthening capacity for natural sciences research: A qualitative assessment to identify good practices, capacity gaps and investment priorities in African research institutions
Source: PLoS One. 2020 Jan 24;15(1):e0228261. doi: 10.1371/journal.pone.0228261 (PMC6980527; doi:10.1371/journal.pone.0228261)
Supplement: S5 Supplementary File — (PDF) [file pone.0228261.s005.pdf]

S5_ Supplementary file 5: Interview guides

Interview Guide for African Principal Investigators

Consent

My name is ***(name)*** and I work with the Capacity Research Unit (CRU) at the Liverpool School of Tropical Medicine. We are working with the Royal Society and DFID to monitor the Africa Capacity Building Initiative. The purpose of this interview is to assist us, the CRU team, to better understand the current capacity of your institution and research consortium to carry out research. We will then use the data obtained from this interview and others to work with you to generate capacity strengthening plans for your institution. It is hoped these capacity strengthening plans will assist in development of individual research capacity and that of your institution. We will also disseminate the research findings internationally through policy briefs and journal articles. With your permission we would like to digitally (audio) record the interview so that we do not miss any information. All information will be held anonymously and securely at all times. You have the right to stop the interview at any time and if there are any questions you do not wish to answer you do not have to. Do I have your permission to continue with the interview? ***(Get the participant to sign the consent form)*** Do you have any questions before we begin?

Introduction to Interview

Thank you for taking the time to meet with me today. I hope you will think of this interview as an opportunity to share your experiences as a principal investigator working within this institution and research consortium. I hope that you feel able to express your opinions openly and honestly so that effective capacity strengthening plans can be developed to ensure the success and sustainability of your institution. Within the interview I will ask you a series of questions based around different topic areas. Firstly, I would like to ask you a few questions about yourself.

General Background

- **Mostly covered in pre-visit questionnaire.**
- **Job role, research focus, role in consortium.**

Strengthening Research Capacity

As I am sure you are aware, one of the main objectives of the Royal Society-DFID African capacity building initiative is to strengthen the research capacity of research institutions and research systems in sub-Saharan Africa, including research uptake and its incorporation into policies and interventions.

- **What do you understand by the term research capacity strengthening?**
- **What do you think ideal research capacity looks like?**
- **Can you tell me about any examples of programmes you know of or have been involved in that have focused on research capacity strengthening?**
    - What worked at the international, national, institutional, individual level?

- Why do you think these methods were effective?
    - What could have been improved about these approaches?
- **How do you think the concept of research capacity strengthening applies to what you do?**

Award Application Process (optional depending time)

Mention that some of this might overlap a little bit with the pre-visit questionnaire, but we want to gain an in-depth understanding of how partnerships were formed and how partners collaborate with each other.

- **Can you tell me how this research consortium formed?**
    - **PROMPT:**
        - Who approached who?
        - Who co-ordinated the inclusion of each partner?
        - Was there a previous network grant? How has it influenced the development of this grant?
- **How were the research questions/key topic areas for this initiative developed?**
    - **PROMPT:**
        - Who led discussions?
        - What level of input did different consortium members have?
        - How well do you feel you and the other partners understand the aims, objectives and methodologies that will be used within the project?

Infrastructure

This will be complemented by our observation checklist

- **What research and study spaces are available at your institution?**
    - **PROMPT:**
        - Computer facilities, quiet study spaces, office space, other spaces, etc.
        - Laboratory facilities: equipment, consumables, space, support from technicians, laboratory collaboration, quality assurance
        - How do you feel these spaces meet the needs of staff and students at your institution?
        - What could be improved about these spaces?
        - What is particularly good about these spaces?

- **How do staff and students access peer reviewed and grey literature?**
    - **PROMPT:**
        - What kind of resources do you have access to?
        - What challenges do you face in accessing literature?
        - What could improve access to literature?
        - What works well when accessing literature?
        - Is there a database for research outputs and projects?

Learning and Teaching

- **What research skills training opportunities are available for students and researchers at your institution? (see questionnaire for additional information)**
  - **PROMPT:**
    - Research methodologies; grant writing and fundraising; monitoring evaluation and planning; managing research and resources; research ethics; intellectual property, writing and disseminating research findings for different audiences; development and nurturing of software skills (computer literacy);
    - Who facilitates research training? (internal/external; national/international)
    - Who accesses research training?
    - How are training needs for students and researchers identified? Once identified, how are training needs addressed?
    - What other research training opportunities would you like to receive?
- **What research training is available to you outside of your institution?**
  - **PROMPT:**
    - What are the benefits of external training?
    - How is this funded?
    - How are these opportunities identified?
- **What research skills training opportunities do you provide at your institution?**
  - **PROMPT:**
    - Who are these for?
    - How are they supported by the institution?
    - How often do these sessions take place?
    - Are these sessions voluntary or compulsory?
    - How equipped do you feel to deliver these sessions?
    - Is there anything that would make delivering these sessions easier?
- **What training is available for non-academic staff?**
- **Are there any departmental seminars/workshops, critical thinking, journal clubs?**
- **What elements of learning and teaching do you particularly enjoy?**
- **What elements of learning and teaching do you feel require improving?**

Mentorship, supervision and guidance

- **To what extent is leadership encouraged at your institution?**
  - **PROMPT**:
    - What evidence can you suggest to show how it is valued?
    - What evidence can you suggest to show how it is not valued?
    - How able do you feel to fulfil a leadership role?
- **What mentorship systems, either formal or informal are in place at your institution?**
  - **PROMPT:**
    - What evidence can you suggest how it is valued?
    - What evidence can you suggest how it is not valued?
    - How able do you feel to fulfil a mentorship role?

- **Can you tell me about the way PhD students are supervised?**
    - **PROMPT:**
        - How many supervisors do students have?
        - Where are they based?
        - Are there minimum standards in place about the level of supervision to be given?
        - What kind of training do supervisors receive?

Organisation and Management

- **What PhD course guidelines are in place for staff and students?**
    - **PROMPT:**
        - PhD handbooks, information about pastoral care, induction courses etc.
        - How is progress throughout the PhD assessed? (e.g. upgrade reports, who sits on the panel etc.)
- **What feedback mechanisms are in place between staff and students? How can students influence processes at the departmental and institutional level?**
    - **PROMPT:**
        - boards of studies
- **What is your understanding of how research priorities are decided upon at your institution?**
    - **PROMPT:**
        - Who is involved in this process?
        - How are research activities recorded at your institution?
        - Is there an institutional/departmental strategic plan? If yes, what are the research priorities?
- **What administrative support is available for research staff at your institution?**
    - **PROMPT:**
        - Financial, logistical, administrative

Financial Management

- **How are research funds (RS/DFID) managed within your institution?**
    - **PROMT:**
        - Who will be responsible for fund allocation and management?
        - Who will be responsible for financial reporting?
        - How do funding amounts compare to actual and predicted costs?
- **How many PhD students do you think you will be able to support within the project?**
- **How will PhD student funds be allocated and administered?**
    - **PROMPT:**
        - Who will manage these funds?
        - What will the funds cover?
            - Stipends, fees, international placements, national placements
        - How do funding amounts compare to actual/ predicted costs?

Partnerships

- **As you're aware, the success of this research consortium is linked to how effectively you and the other principal investigators work together. Can you tell me, in your experience what makes a successful research partnership?**
    - **PROMPT:**
        - Communication, clear roles and responsibilities, previous relationships
- **What do you think are the benefits to being part of the consortium?**
    - **PROMPT:**
        - Benefits to UK partner, benefits to other southern partners

REVIEW RESPONSES FROM QUESTIONNAIRE BEFORE ASKING THE BELOW, TAILOR QUESTIONS ACCORDINGLY.

- **What do you see as your institutions main strengths in postgraduate training and conducting research?**
- **What do you see as your institutions main weaknesses in postgraduate training and conducting research?**
- **How do you think these weaknesses can be improved?**

Thank you very much for answering all of our questions, is there anything else that you would like to add?

PhD Supervisor Topic Guide

Consent

My name is ***(name)*** and I work with the Capacity Research Unit at the Liverpool School of Tropical Medicine. We are working with the Royal Society and DFID to monitor research capacity strengthening progress of the Africa Capacity Building Initiative. The purpose of this interview is to assist us, the CRU team, to better understand the current capacity of your institution to carry out research, particularly in relation to facilitating students. We will then use the data obtained from this interview and others to generate capacity strengthening plans for your institution. It is hoped these capacity strengthening plans will assist in development of research capacity at your institution. We will also disseminate the research findings internationally through policy briefs and analysis, and journal articles. With your permission we would like to digitally (audio) record the interview so that we do not miss any information. All information will be held anonymously and securely at all times. You have the right to stop the interview at any time and if there are any questions you do not wish to answer you do not have to. Do I have your permission to continue with the interview? ***(Get the participant to sign the informed consent form)*** Do you have any questions before we begin?

Introduction to Interview

Thank you for taking the time to meet with me today. I hope you will think of this interview as an opportunity to share your experiences as a PhD student supervisor within this institution and research consortium. I hope that you feel able to express your opinions openly and honestly, so that effective capacity strengthening plans can be developed to ensure the success and sustainability of your institution. Within the interview I will ask you a series of questions based around various topics. Firstly, I would like to ask you a few questions about yourself.

General Background and Demographics

- What is the role of your institution?
- What is your role within the institution?
    - Do you have any additional roles?
- How long have you been in this (these) role(s)?
- What is your highest level of academic qualification?
- What is your involvement with the Royal Society or this initiative beyond this research?
- What is your area of specialisation?
- What is your first language?
- Can you speak any other languages fluently?
- How old are you?

Strengthening Research Capacity

You may or may not be aware, that one of the main objectives of the Royal Society-DFID African capacity building initiative is to strengthen the research capacity of research institutions and research systems in sub-Saharan Africa, including research uptake and its incorporation into policies and interventions.

- **What do you understand by the term capacity strengthening?**
- **What do you think ideal research capacity looks like?**
- **Can you tell me about any examples of programmes you know of that have focused on research capacity strengthening?**
  - **What worked at the international, national, institutional, individual level?**
  - **Why do you think these methods were effective?**
  - **What could have been improved about these approaches?**
- **How do you think the concept of research capacity strengthening applies to what you do?**

Student Allocation and Funding

- How many PhD students have you supervised/are you currently supervising?
  - Is your PhD student's (supported by the Award) thesis in your area of expertise
  - Do you supervise students external to this institution/department?
- **How are you allocated PhD students within your department/institution?**
  - How did this process work for the student funded by the Royal Society-DFID initiative?
- **How are you incentivised to supervise PhD students?**
- **How are PhD students normally funded at your institution?**
  - **PROMPT:**
    - Who manages these funds?
    - What do the funds cover?
      - Stipends, fees, international placements, national placements
    - How do funding amounts compare to actual costs?
    - If relevant, how does the funding process differ for your Royal Society students?

Mentorship, Supervision and Guidance

- **According to you, can you tell me what makes a good PhD supervisor?**
- **How are PhD students inducted at your institution/department?**

  PROMPT:

  - PhD handbook, induction lectures, orientation tours
  - Based on your experience what do you think helped the students the most?
  - What do you feel could be improved about the induction process?

How do you support your students to identify their training needs?

- **How are PhD students training needs met at your institution? (e.g. research methodology course; research ethics; paper writing; grant writing etc.)**

  Can you tell me about the way your PhD students are supervised?
  - **PROMPT:**
    - How many supervisors do the students have?
    - Where are your students based?
    - How often do you meet with them?
    - How often do you communicate (phone, email) with them?
    - Are there minimum standards in place about the level of supervision you are provide?
    - How does the supervision process of the RS/DFID-funded student differ from self-funded PhD students
- **How are your students encouraged to interact with other PhD students?**
- **What mentorship systems, either formal or informal are in place at your institution?**
  - **PROMPT:**
    - Other than you, what other senior researchers are available to provide support and guidance to your students?
    - How would you evaluate the mentorship process for the professional learning and development of your students?
    - What elements of the mentorship process are particularly beneficial?
    - What elements of the mentorship process do you feel could be improved?
- **How is progress of your PhD student monitored?**
  - **PROMPT:**
    - How often is progress monitored?
    - What is the process if they are not progressing as expected?
    - What guidelines are in place in relation to monitoring progress?

Research Uptake

- **Once your students have completed their research, how do you encourage them to disseminate the findings?**
  - **PROMPT:**
    - Publication, policy briefings, lay summaries, stakeholders meetings, blogs, twitter, other social media.

Final questions

- **What do you see as your institutions three main strengths in conducting research?**
- **What do you see as your institutions three main weaknesses in conducting research?**
- **How do you think these weaknesses can be improved?**

Thank you very much for answering all of our questions, is there anything else that you would like to add?

Interview Guide for PhD Students

NB to Interviewer- depending on the time at which you are interviewing the research student and their previous affiliation to the institute/consortia they may or may not be able to answer some of the questions within this guide. It is up to the interviewer to use their judgement to get as much relevant information as possible.

Introduction

- **Role of CRU within the RS/DFID initiative**
  - Brief background on Initiative and description of consortia
  - Highlight CRU's role as a broker between RS/DFID and consortia
- **Baseline assessment**
  - Purpose
  - People involved
  - Themes covered
  - Overview of key findings
  - Action planning

General Background (if not already captured)

Understanding of Research Capacity Strengthening

You may or may not be aware, that one of the main objectives of the Royal Society-DFID African capacity building initiative is to strengthen the research capacity of research institutions and research systems in sub-Saharan Africa, including research uptake and its incorporation into policies and interventions.

- **What do you understand by the term capacity strengthening?**
- **What do you think ideal research capacity looks like?**
- **Can you tell me about any examples of programmes you know of or you were involved in that have focused on research capacity strengthening?**
  - **What worked well and what didn't? Why?**

Application, Admissions and Funding

- **How did you register for this PhD?**
  - **PROMPT:**
    - Who
    - How long did it take?
    - What challenges did you face?
- **Once you had been accepted to study and started your programme, what information were you given?**
  - **PROMPT:**
    - PhD handbook, induction lectures, orientation tours
    - What information did you find useful?

            - What information would you have liked to receive and didn't?
- **How will your studies be funded?**
    - **PROMPT:**
        - Who will manage these funds?
        - What will the funds cover?
            - Stipends, fees, international placements, national placements, labtops
        -
        - What are your research costs?
- Do you think your experience differs from other PhD students within this institute/department? If, yes how?

Infrastructure

- **What research facilities are available at your institution?**
    - **PROMPT:**
        - Computer facilities, lecture halls, quite study spaces, office space etc
        - Laboratory facilities:
            - (External) Quality assurance systems are in place?
            - What equipment is there? What equipment is missing? How is equipment procured?
            - What laboratory- related technical support and skills training do you receive?
            - Is there a health and safety system in place?
            - How do you network with other laboratories?
        - How do you feel these spaces meet the needs of students at your institution?
        - What could be improved about these spaces?
        - What is particularly good about these spaces?
        - What resources would make studying easier?

- **How do staff and students to access peer reviewed and grey literature?**
    - **PROMPT:**
        - What literature do you have access to?
        - What challenges do you face in accessing literature?
        - What could improve access to literature?
        - What works well when accessing literature?

Learning and Teaching

- **What research skills training is available for you at your institution?**
    - **PROMPT:**
        - Research methodologies; grant writing and fundraising; monitoring, evaluation and planning; managing research and resources; research ethics; intellectual property; writing and disseminating research findings for different audiences; IT- developing and nurturing of software skills (computer literacy)
        - How are training needs identified?

        - How are your training skills assessed?
- **What research skills training is available to you outside of this institution?**
    - **PROMPT:**
        - Other institutions, national, international
        - How are these funded?
        - How are these identified?
- **What other training opportunities would you like to receive and why? How do you go about receiving these training opportunities?**

Mentorship, Supervision and Guidance

- **Can you tell me about the way your PhD is supervised?**
    - **PROMPT:**
        - How many supervisors do you have for your PhD?
        - Where are they based?
        - How often do you meet with them?
        - How often do you communicate (phone, email) with them?
        - What is their area of specialisation?
        - What is their experience in your area of research?
        - How has this influenced your experience at the institution?
        - Are there minimum standards in place about the level of supervision you are to be given?
- **What interaction do you have with other students and in wider consortium?**
    - What facilitates this?
        - Seminars? Common rooms? Shared working spaces? Social events?
    - How does this influence your learning?
- **What mentorship systems, either formal or informal are in place at your institution?**
    - **PROMPT:**
        - Other than your supervisor, what other senior researchers are available to you to provide support and guidance?

Organisation and Management

- **How is progress in your PhD monitored?**
    - **PROMPT:**
        - How often is progress monitored? Who monitors progress?
        - What is the process is you are not progressing as expected?
        - What guidelines are in place in relation to monitoring progress?
- **What feedback mechanisms are in place between staff and students?**
    - **PROMPT:**
        - Class representatives, boards of studies, how are students represented?

Final questions

- **What do you see as your institutions main strengths of postgraduate training and research?**

- **What do you see as your institutions main weaknesses of postgraduate training and research?**
- **How do you think these weaknesses can be improved?**
- **What are your career plans on completion of your studies?**

Thank you very much for answering all of our questions, is there anything else that you would like to add?

Interview Guide for Heads of Department/Institute Deans or Principles

Consent

My name is ***(name)*** and I work with the Capacity Research Unit (CRU) at the Liverpool School of Tropical Medicine. We are working with the Royal Society and DFID to monitor the research capacity strengthening progress of the Africa Capacity Building Initiative which funds a research consortium which staff at your institute are involved with. The purpose of this interview is to assist us to better understand the current capacity of your institution to carry out research. We will then use the data obtained from this interview and others to generate capacity strengthening plans for your institution. It is hoped these capacity strengthening plans will assist in development of research capacity at your institution. We will also disseminate the research findings internationally through policy briefs and analysis, and journal articles. With your permission we would like to digitally (audio) record the interview so that we do not miss any information. All information will be held anonymously and securely at all times. You have the right to stop the interview at any time and if there are any questions you do not wish to answer you do not have to. Do I have your permission to continue with the interview? ***(Get the participant to sign the consent form)*** Do you have any questions before we begin?

Introduction to Interview

Thank you for taking the time to meet with me today. I hope you will think of this interview as an opportunity to share your institutes' expertise. I hope that you feel able to express your opinions openly and honestly so that effective capacity strengthening plans can be developed to ensure the success and sustainability of your institution. Within the interview I will ask you a series of questions based around different topic areas. Firstly, I would like to ask you a few questions about yourself.

General Background and Demographics

- What is the role of your institution?
- What is your role within the institution?
    - Do you have any additional roles?
- How long have you been in this (these) role(s)?
- What is your involvement with the Royal Society or this initiative beyond this consortium grant?
- What is your area of specialisation?
- What is the highest level of academic qualification you have obtained?
- What is your first language?
- Can you speak any other languages fluently?

Strengthening Research Capacity

You may or may not be aware that one of the main objectives of the Royal Society-DFID African capacity building initiative is to strengthen the research capacity of research institutions and research systems in sub-Saharan Africa, including research uptake and its incorporation into policies and interventions.

- **What do you understand by the term capacity strengthening?**
- **What do you think ideal research capacity looks like?**
- **Can you tell me about any examples of programmes you know of or have been involved with that have focused on research capacity strengthening?**
    - What worked at the international, national, institutional, individual level?
    - Why do you think these methods were effective?
    - What could have been improved about these approaches?
- **How do you think the concept of research capacity strengthening applies to what you do?**

Infrastructure

- **What research and study spaces are available at your institution?**
    - **PROMPT:**
        - Computer facilities, quiet study spaces, office space, other spaces etc.
        - Laboratory facilities:
            - Quality assurance systems are in place?
            - What equipment is there? What equipment is missing? How are equipment and consumables procured?
            - Is there a health and safety system in place?
            - How do you network with other laboratories?
        - How do you feel these spaces meet the needs of staff and students at your institution?
        - What could be improved about these spaces?
        - What is particularly good about these spaces?

- **How do staff and students to access peer reviewed and grey literature?**
    - **PROMPT:**
        - What challenges do you face in accessing literature?
        - What could improve access to literature?
        - What works well when accessing literature?

Learning and Teaching

- **What research skills training opportunities are available for staff at your institution?**
    - **PROMPT:**
        - Research methodologies; grant writing and fundraising; monitoring evaluation and planning; managing research and resources; research ethics;

writing and disseminating research findings for different audiences; development and nurturing of software skills (computer literacy)
        - How are training needs identified?
        - What other research training opportunities would you like them to receive?
- **What management skills training opportunities are available to staff at your institution?**
    - **PROMPT:**
        - Supervision and mentorship, grant management, financial management,
        - How are training needs identified?
        - What other management skill training opportunities would you like to receive?
- **What research training is available to staff outside of your institution?**
    - **PROMPT:**
        - How is this funded?
        - How are these opportunities identified?
- **What management training is available to staff outside of your institution?**
    - **PROMPT:**
        - How is this funded?
        - How are these opportunities identified?
- **Can you tell about how are students managed at your institution?**
    - **PROMPT:**
        - Who is responsible for this?
        - What supervision structures are in place?
        - What assessment guidelines exist?
        - What are the staff-student feedback mechanisms?
        - How does the institute work with external examiners?

Mentorship, Supervision and Guidance

- **To what extent is leadership encouraged at your institution?**
    - **PROMPT**:
        - What evidence can you suggest as to how it is valued?
        - What evidence can you suggest as to how it is not valued?
        - How able do you feel to fulfil a leadership role?
- **What mentorship systems, either formal or informal are in place at your institution?**
    - **PROMPT:**
        - What evidence can you suggest as to how it is valued?
        - What evidence can you suggest as to how it is not valued?
        - How able do you feel to fulfil a mentorship role?
- **What is the research environment like at your institution?**
    - **PROMPT:**
        - How is it supportive/active?
        - Where could it be improved?
        - What evidence do you have of this?

Organisation and Management

In assessing your institutes' capacity, it is important to examine the organisation and strategic planning of the institute. We are particularly interested in learning about the strengths as well as weaknesses in the organisation of the institution.

- **Does your institute/department have a strategic plan?**
  If yes;
  - **Does this plan include research? Is there a separate strategic plan in place specifically for research?**
  - **What is your view of the strategic plan?**
  - **How is it used in planning and development?**
  - **To what extent does the strategic plan of your institute/department align with institutional, national and international research priorities?**
  - **How are you involved in developing and implementing the plan?**

- **How are research priorities and topics decided upon?** (*This question may depend on the level of the person being interviewed- those at the top level may not know how this is done on a departmental basis)*
  - **PROMPT:**
    - Who is involved in this process?
    - How are research activities recorded at your institution?

- **If relevant, how is research ethics review conducted at your institution?**
  - **PROMPT:**
    - What guidelines are in place to guide researchers through the ethics process?
    - What guidelines are in place relating to academic honesty and plagiarism?

- **Can you tell me about how human resources are managed at your institution?**
  - **PROMPT:**
    - How are staff recruited?
    - How is staff promotion and progression managed?
    - How fair do you think individuals salaries are based on their roles and responsibilities?
      - How do these compare to other institutes in your country/network?
    - What policies are in place relating to HR?
    - What role does HR play in supporting research?

Financial Management

- **What are the main funding sources for your institution?**
  - **PROMPT:**
    - Public national funds, private national funds, international grants, core funds
- **How are financial resources managed at your institution?**
  - **PROMPT:**

        - Who manages them?
        - What financial reporting regulations are adhered to?
        - What policies and procedures are in place that relate to finance?
        - How is transparency insured?
        - How is financial sustainability insured?
        - How are resources split between research and operations?
        - How are overheads managed?

Partnerships

The consortium with which your institution is involved involves two other African partners and a UK based partner.

- **Can you tell me, in your experience what makes a successful research partnership?**
    - **PROMPT:**
        - Communication, clear roles and responsibilities, previous relationships

- **What links does your institution have with other institutions?**
    - **PROMPT:**
        - National, international, public, private, research, non- research, joint posts

- **How do you feel that your institute can benefit from being part of this research consortium?**
    - **PROMPT:**
        - Knowledge exchange, financial, infrastructure, prestige, sustainable partnerships,

Research Uptake

- **How does your institution disseminate research findings?**
    - **PROMPT:**
        - Publications, seminars, annual report, conferences, policy briefings, stakeholder meetings, blogs, twitter, other social media
- **Can you comment on consortia links to national and international policy makers?**
- **What are the mechanisms by which you think that the research conducted by this consortium may influence policy decisions?**
- **Can you list the national and international research networks to which your consortium is linked?**
    - **What is your institution/departments role within these networks?**

Final questions

- **What do you see as your institutions main strengths in postgraduate training and conducting research?**
- **What do you see as your institutions three main weaknesses in postgraduate training and conducting research?**
- **How do you think these weaknesses can be improved?**

Thank you very much for answering all of our questions, is there anything else that you would like to add?

Interview Guide for Award/Institute Support Staff

Consent

My name is ***(name)*** and I work with the Capacity Research Unit at Liverpool School of Tropical Medicine. We are working with the Royal Society and DFID to monitor research capacity strengthening progress of the Africa Capacity Building Initiative. The purpose of this interview is to assist us, the evaluation team, to better understand the current capacity of your institution and research consortium to carry out research. We will then use the data obtained from this interview and others to work with the project principal investigator to generate capacity strengthening plans for your institution. It is hoped these capacity strengthening plans will assist in development of individual capacity and that of your institution. We will also disseminate the research findings internationally through policy briefs and analysis, and journal articles. With your permission we would like to digitally (audio) record the interview so that we do not miss any information. All information will be held anonymously and securely at all times. You have the right to stop the interview at any time and if there are any questions you do not wish to answer you do not have to. Do I have your permission to continue with the interview? ***(Get the participant to sign the consent form)*** Do you have any questions before we begin?

Introduction to Interview

Thank you for taking the time to meet with me today. I hope you will think of this interview as an opportunity to share your experiences as an administrator working within this institution and research consortium. I hope that you feel able to express your opinions openly and honestly so that effective capacity strengthening plans can be developed to ensure the success and sustainability of your institution. Within the interview I will ask you a series of questions based around different topic areas. Firstly, I would like to ask you a few questions about yourself.

General Background and Demographics

- What is the role of your institution?
- What is your role within the institution?
    - Do you have any additional roles?
- How long have you been in this (these) role(s)?
- What is your involvement with the Royal Society or this initiative beyond this consortium grant?
- What is your area of specialisation?

Human Resources (Human Resource Office)

- **Can you tell me about human resource management at your institution?**
    - **PROMPT:**
        - How are staff recruited?
        - What staff appraisal systems are in place?

        - How is staff promotion managed?
        - How fair do you think individuals salaries are based on their roles and responsibilities?
            - How do these compare to other institutes in your country/networks?
        - What policies are in place relating to HR?
            - Maternity leave, gender equity, disciplinary procedures
        - Do you have a job description?
            - If yes, how well does your job description match your roles and responsibilities?
            - If yes, how do you think this fits with institutional aims and objectives?
            - If no, how well do you feel you understand what is expected of you to complete your job role?
- **Can you tell me about the administrative systems and guidelines that are in place at your institution?**
    - **PROMPT:**
        - What research management systems exist?
        - How are research activities recorded?
        - How are responsibilities delegated?
        - How clear and understandable are systems?
        - What could improve these systems?
        - What works well about these systems?
- **What are your office's main strength with regard to facilitating postgraduate training and research?**
- **What are your office's main weaknesses with regard to facilitating postgraduate training and research?**
- **How can the weaknesses be addressed?**

Financial Management (Finance Office)

- **How are financial resources managed at your institution?**
    - **PROMPT:**
        - Who manages them?
        - What financial reporting regulations are adhered to?
        - What policies and procedures are in place that relate to finance?
        - How is transparency ensured?
        - How is financial sustainability ensured?
        - How are resources split between resources and operations?
- **How could financial management systems be improved at your institution?**
    - **PROMPT:**
        - How could guidelines be made clearer?
        - What other systems could be put in place?
        - What training could be offered to staff?

- **What are your office’s main strength with regard to facilitating postgraduate training and research?**
- **What are your office’s main weaknesses with regard to facilitating postgraduate training and research?**
- **How can the weaknesses be addressed?**

Research office

- **Can you tell me about the role of the Research Office at your institutions?**
  - **PROMPT:**
    - How are research priorities decided upon and who is involved?
    - How are funding opportunities identified?
    - What are the main funding sources at your institution?
    - What kind of research collaborations exists between your institution and external partners (national and international; research and industry)?
    - What kind of support do researchers receive from your office?
- **What are your office’s main strength with regard to facilitating postgraduate training and research?**
- **What are your office’s main weaknesses with regard to facilitating postgraduate training and research?**
- **How can the weaknesses be addressed?**

Graduate School

- **Can you tell me about the role of the Research Office at your institutions?**

Application, Admissions and Funding

- **How do students register for a PhD?**
  - **PROMPT:**
    - What/who is involved in the process?
    - How long does it take?
    - How do you evaluate this process?
- **Once the student is accepted to study and starts the programme, what information is he/she given?**
  - **PROMPT:**
    - PhD handbook, induction lectures, orientation tours
    - How do you evaluate the process
- **How will your studies be funded?**
  - **PROMPT:**
    - Who will manage these funds?
    - What will the funds cover?
      - Stipends, fees, international placements, national placements

        - How do funding amounts compare to actual costs?
        - What are your research costs?
- Do you think your experience differs from other PhD students within this institute/department? If, yes how?

Laboratory staff

- **Can you tell me about the laboratories (teaching and research) in your department/institution?**
    - **PROMPT:**
        - How many laboratories for teaching and how many for research?
        - How are the laboratories used and accessed?
        - What kind of equipment is there in the laboratories? Is it sufficient?
        - How is the equipment maintained?
        - Does the laboratory adhere to quality assurance system?
        - How are experiments logged? Laboratory log books?
        - How are students/staff trained to use the laboratories (induction, health and safety)? Is the training sufficient?
        - Can you describe the procurement process?
        - How is health and safety managed?
        - How are laboratory services promoted?
        - Do you collaborate with other laboratories (internal/external)?
- **What are the laboratories' main strength in terms of postgraduate training and research?**
- **What are the laboratories' weaknesses in terms of postgraduate training and research?**
- **How can the weaknesses be addressed?**

Thank you very much for answering all of our questions, is there anything else that you would like to add?
