# Supplementary material for: Strengthening capacity for natural sciences research: A qualitative assessment to identify good practices, capacity gaps and investment priorities in African research institutions
Source: PLoS One. 2020 Jan 24;15(1):e0228261. doi: 10.1371/journal.pone.0228261 (PMC6980527; doi:10.1371/journal.pone.0228261)
Supplement: S6 Supplementary File — (DOCX) [file pone.0228261.s006.docx]

### S6_ Supplementary file 6: Thematic framework used to analyse research capacity strengths and gaps

|  | **Thematic Areas** | | | | | | | | | |
| --- | --- | --- | --- | --- | --- | --- | --- | --- | --- | --- |
| **Position** | **Research infrastructure and facilities** | **Learning and Teaching** | **Research strategies and support** | **PhD programme** | **Financial management and funding** | **Collaborations and partnerships** | **Main strengths** | **Other** | **Key quotes from interviews** | **Important info from questionnaire** |
| **PI (co-PI)** |  |  |  |  |  |  |  |  |  |  |
| **HoD** |  |  |  |  |  |  |  |  |  |  |
| **Dean** |  |  |  |  |  |  |  |  |  |  |
| **PhD** |  |  |  |  |  |  |  |  |  |  |
| **Chief laboratory technician** |  |  |  |  |  |  |  |  |  |  |
| **Graduate School** |  |  |  |  |  |  |  |  |  |  |
| **Research Office** |  |  |  |  |  |  |  |  |  |  |
| **SUMMARY** |  |  |  |  |  |  |  |  |  |  |

Sub-areas were captured for each thematic area and highlighted in bold. These varied depending on the institution. New thematic areas and sub-areas inevitably arose during the data collection process and the analysis frameworks were adapted accordingly
