# Supplementary material for: Strengthening capacity for natural sciences research: A qualitative assessment to identify good practices, capacity gaps and investment priorities in African research institutions
Source: PLoS One. 2020 Jan 24;15(1):e0228261. doi: 10.1371/journal.pone.0228261 (PMC6980527; doi:10.1371/journal.pone.0228261)
Supplement: S7 Supplementary File — (DOCX) [file pone.0228261.s007.docx]

### S7_ Supplementary file 7: Strengths and gaps in research capacity that emerged from the data for each theme, with examples of good practices and ways of solving problems that may be useful to other institutions.

**S7_Table 1: Research infrastructure strengths - assessment findings**

| **Institution synonym** | **Office space** | **IT** | **Library and literature** | **Laboratories** |
| --- | --- | --- | --- | --- |
| R1A | - Sufficient | - Campus-wide Wi-Fi - Competent computer centre offering support and training - ICT policy development - High Performance Computer (HPC) - Adequate computer laboratories | - Well-equipped library in terms of space and literature - Department-specific libraries - Library assists with getting articles from journals the library is not subscribed to - Access to Research4Life e-database (AGORA) | - Basic equipment - Qualified laboratory technician - Existing collaboration with other laboratories |
| R1B | - Sufficient (work space in shared office and in doctoral school) | - Mostly reliable internet (government-funded rapid internet access) | - National plans to extend e-learning connecting resources of all universities in country - Part of Research 4 Life | - Laboratory offers services to internal and external users, charging bench fees. The bench fees are reinvested in laboratory |
| R1C | - No specific strengths reported | - HPC | - Access to most relevant literature | - Department has access to external laboratories for some work (fee is charged) - State of equipment is regularly reported to department |
| R1D | - No specific strengths reported | - No specific strengths reported | - Access to most relevant literature - Access to Research 4 Life (AGORA) | - Started new quality assurance programme as part of the AGRA programme - Field sites for data collection are not secure 🡪 can affect the data quality if field site is tampered with |
| R1E | - PhD students normally work in common room | - No specific strengths reported | - Access to key/basic journals | - Laboratories are equipped with most crucial equipment - Laboratory offers services to internal and external users, charging bench fees - Adequate health and safety procedures/standards in place |
| R1F | - No specific strengths reported | - Good access to internet and IT - Department has well equipped computer lab and there is a computer lab for postgraduate student at the university level | - Adequate access to most literature - Library offers courses - Library facilitates access to unsubscribed articles | - Laboratories have very good national and international reputation - Income from offering laboratory services is re-invested in laboratory - Sell laboratory manuals to students. Income is re-invested in the laboratory infrastructure - Induction for new students - Adequate health and safety process |
| R2A | - Dedicated office space for research team working on RS project | - Adequate access to computers with relevant software installed on them | - No specific strengths reported | - No specific strengths reported |
| R2B | - Adequate office space - Common room to work in | - IT facilities and services adequate - There is an IT section in the institution | - Own institutional library - Access to relevant online data bases | - Laboratories adequately equipped to do most of the research - Effective equipment maintenance system - Effective laboratory networking and use of laboratory services by external partners - QA and H&S systems are in place |
| R2C | - Each PhD student has a work space with computer | - Very good IT facilities and IT support | - Very well resourced library - Training courses offered by library - Access to online journals | - Very well equipped laboratories with state-of-the-art equipment |

### S7_Table 2: Research infrastructure gaps and challenges - assessment findings

| **Institution synonym** | **Office space** | **IT** | **Library and literature** | **Laboratories** |
| --- | --- | --- | --- | --- |
| R1A | - No challenges reported | - Slow broadband speed (especially during day term times) - Purpose of HPC unclear to many participants | - Resources in library often outdated - Inconsistent knowledge about which literature/database is accessible | - Most equipment outdated (no major equipment has been bought for 10 years); needs maintenance and repair - Lack of funding for equipment and training - Collaboration with other laboratories expensive - Lack of SOPs and reference material - Limited laboratory space due to high numbers of students. Affecting quality of research - Inadequate quality assurance system - Need for effective laboratory management information and archival system - Inadequate health and safety standards/equipment; need for training |
| R1B | - Some space issues as students sometimes sit in supervisor’s office | - No challenges reported | - No challenges reported | - Difficult procurement processes |
| R1C | - Insufficient working space for PhD students | - Frequent power shortages affect research - Inadequate access to the internet, lack of necessary bandwidth to download data - Lack of departmental IT staff | - Lack of communication on what support library can offer to postgraduate students - Difficulty to get some ‘classic’ literature - Inconsistent knowledge about which literature/database is accessible | - Lack of state of the art equipment - Laboratory technicians lack training to repair and maintain equipment - Differences in access to equipment among self-funded and grant-funded students (e.g. self-funded students pay for chemicals themselves) - Difficult procurement processes - Need to improve quality assurance systems |
| R1D | - Inadequate working space in laboratory | - Internet is intermittent and lacks relevant bandwidth - Frequent power shortages | - Lack of communication on what support library can offer to postgraduate students | - Sharing of facilities with undergraduate students 🡪 affects research quality - Difference between self- and grant-funded students - Crucial equipment is outdated and/or lacking 🡪 forced to use out of date research methodology, which is time-consuming - Laboratory technicians need training to maintain and repair equipment - Lack of funding for equipment, maintenance and repair - Need to improve health and safety procedures (training of lab techs on H&S) - Lab techs needs are overlooked when it comes to training and promotion |
| R1E | - Need to enlarge work spaces for research students | - Frequent power cuts affect research | - Lack of access to relevant journals/ lack of relevant subscriptions | - Some special research equipment lacking - Laboratory techs need training especially in handling certain equipment - Difficult procurement processes |
| R1F | - Space issues for PhD students due to increasing number of undergraduate students | - No challenges reported | - No challenges reported | - Institutional funds for maintenance and repair are relatively low - Procurement of consumables can be lengthy (but no challenges with customs) |
| R2A | - No challenges reported | - Internet is intermittent and lacks relevant bandwidth - Frequent power shortages 🡪 very challenging for computational part of the research | - Lack of access to relevant journals/lack of relevant subscriptions (main challenge) - Inconsistent knowledge about which literature/database is accessible | - Lack of crucial equipment - Lack of funding for equipment - Lab quality assurance needs strengthening - Lack of crucial consumables - Need for training of laboratory technicians |
| R2B | - No challenges reported | - Frequent power shortages affect research - Internet access not always reliable and some scientists use their own modems to ensure access to the internet - No institutional wide mailing system (to address this gap was one main priority) | - Need for more subscriptions to resources to become centres of excellence | - Some crucial equipment is outdated - Space issues – need to enlarge work space - Due to space issues, some samples could not be stored and needed to be destroyed, which affects the research quality assurance |
| R2C | - No challenges reported | - No challenges reported | - No challenges reported | - As laboratory services are used by different internal and external stakeholders, there are sometimes long waiting times to use certain equipment/conduct certain analysis - Only researchers maintain the equipment |

### S7_Table 3: Summary of innovative or good practice, and problem solving: research infrastructure

|  | **Suggestions** | **Good practice** |
| --- | --- | --- |
| **Workspace** | - Separate teaching and research laboratories - Allocation of specific working spaces for postgraduate students, particularly PhD students. If space issues remain, the department could develop timetables that indicate which student group (either postgraduate or undergraduate students) uses the laboratory space at specific dates and times |  |
| **ICT** | - Purchase of generators/solar panel for reliable electricity supply - Institutional IT service to offer training on basic trouble shooting of PCs and laptops - Consortia should also explore possibilities of sharing license agreements for certain software - Need to communicate funding for PhD students more effectively as not all students aware what is included in their stipend in terms of equipment (e.g. laptop) - Establishment of effective institutional email system, which will facilitate institutional communication |  |
| **Library/ Literature** | - Communicate access to resources (e.g. Research4Life) across consortia during meetings attended by all consortia - Establish a list of key journals and time frame that the department should pursue funding for - Communicate library services and trainings more effectively to staff and students, for instance through emails to all staff by the library team | - Development of effective strategies to obtain articles through use of specific networks |
| **Laboratories** | - Communicate purchase of new equipment (purpose, target audience) to all staff and students through online communication and notice boards - Share the ACBI guidelines and budget with whole research team - Develop effective marketing activities and offering laboratory services to internal and external stakeholders - ISO accreditation as main strategy to market laboratory services - Include costs for training of lab techs, equipment repairs and maintenance more effectively into funding proposals - Market laboratory services sell manuals to students and reinvest money into laboratory - Some crucial equipment needed for the project has not been budgeted for. This can be addressed by organising meetings with all stakeholders (PIs, lab technicians, students) discussing available equipment and equipment needs (development of laboratory inventory) of each partner to make smart purchasing decisions - If possible, explore possibilities of purchasing equipment by institutions with more efficient procurement systems and then share equipment across partners - Department to develop mechanisms that ensure equitable access to equipment and laboratory space for all PhD students (including self-funded students) - Develop a laboratory inventory and ‘needs assessment’ of equipment relevant to research areas identified within the strategic plan and systematically include the needed equipment in proposals for funding - Develop more formalised system for procurement - Funding efforts should be extended to include health and safety equipment within the laboratories | - Selling laboratory manuals to undergraduate students and investing the generated income in the laboratory infrastructure and in postgraduate training - Use consortium inception meetings to discuss ways to utilise project-funded equipment most effectively. Development of laboratory inventories across all partner institutions to make smart decisions about purchase of equipment |

### S7_Table 4: Learning and teaching strengths - assessment findings

| **Institution synonym** | **Training for academic staff** | **Training for students** | **Training for non-academic staff** |
| --- | --- | --- | --- |
| R1A | - Some institutional funding for training of academic staff, though limited | - Training needs are assessed by supervisors - Mandatory research skills training courses organised by the Postgraduate Centre - Use people from governments as supervisors who can offer training in themes relevant to national needs | - No specific strengths reported |
|  | There is a quality assurance guidance document on learning and teaching and a Learning and Teaching Centre that aims at ensuring teaching and learning excellence in the university | | |
| R1B | - University-wide research skills training courses for staff and students - Doctoral schools in the process of enlarging their training courses to include other academic staff beyond research students - Government funding for training abroad every other year - Academic staff expected to complete training in pedagogy - Recent focus on leadership and management training for senior academics (conducted by external organisation) | - Supervisors required to assess training needs of PhD students - Contract between supervisor and students ensuring that needed trainings are conducted effectively - PhD students have the opportunity to teach and some level of supervision during field works or industrial attachments | - No specific strengths reported |
| R1C | - No specific strengths reported | - Postgraduate training courses in research methods offered at institutional level - Department offers courses in presentation skills and literature review | - No specific strengths reported |
| R1D | - Training in academic writing, grant writing, ethics and presentation skills available at institutional level | - Subject-specific training offered at departmental level - Self-funded students have benefitted from training that grant-funded students received organised by the research programme | - No specific strengths reported |
| R1E | - Training needs assessment take place during line management meetings and training approved by the HoD (line management meetings are no longer compulsory) - Government funding for writing retreat weekends | - Mandatory generic skill training courses at the beginning of postgraduate studies - Informal training needs assessment with supervisor - Institutional generic skills training courses available in academic writing. Also, formal guidelines on how to write a research proposal from the Research and Innovation Office - Funds for training of postgraduate students available at departmental level - Government funding for writing retreat weekends | - No specific strengths reported |
| R1F | - Institutional funding to attend conferences - No formal training needs assessment for academic staff - Academic staff encouraged to take research leave to attend training courses - Training is included in annual budgets | - Training needs assessment done by PhD supervisor - Some (but little) funding for training either from department or project funds from supervisor - All PhD students have access to generic skill training courses offered through the School of Graduate Studies and the Office of Research and Development - Departmental seminars important for learning - Institutional funding to attend conferences - Training is included in annual budgets | - Certain proportion of funding for non-academic staff, including laboratory staff (especially when new equipment is purchased) - Training is included in annual budgets |
| R2A | - Regular departmental, faculty and institutional meetings where academics’ and students’ training needs are discussed - Short institutional courses for all academic staff | - Regular departmental, faculty and institutional meetings where academics’ and students’ training needs are discussed | - No specific strengths reported |
| R2B | - Training courses for staff and students frequently offered nationally and internationally - Academic staff discuss training needs with line manager - Weekly seminars open to all staff - Teaching loads considerably lower than at high education institution, so most of academics’ time is dedicated to research | - Training needs assessment between student and supervisor, where both decide which skills to focus on - Mandatory courses for PGR students at the institutional level: science communication, statistics, knowledge management - Training courses for staff and students frequently offered nationally and internationally - Weekly seminars open to all staff and students | - Non-academic staff discusses training needs with line manager - Training courses for staff and students frequently offered nationally and internationally - Female staff particularly encouraged to participate in training - Weekly seminars open to all staff |
| R2C | - Training needs discussed with the line manager every year (every 6 months, staff is evaluated) - Every 6 months, staff has to set him/herself key performance indicators which will be reviewed/checked with line manager - Wide range of training courses available for research staff at the institutional level. Some are free and some incur a fee - Funding for training comes from research fund | - Wide range of training courses offered, which are not compulsory - Student normally discusses training needs with supervisors | - Training needs assessment done for non-academic staff - Skill training courses available for non-academic staff |

### S7_Table 5: Learning and teaching gaps and challenges - assessment findings

| **Institution synonym** | **Training for academic staff** | **Training for students** | **Training for non-academic staff** |
| --- | --- | --- | --- |
| R1A | - Training needs of academic staff not formally assessed - Need for training in project and financial management (especially international grants management) - Limited institutional and governmental funding for training - High teaching loads of academic staff | - No formal guidelines on how training needs should be assessed - Need for more training in SPSS, GIS, ‘R’, GenStat, academic writing, writing funding proposals - Limited institutional and governmental funding for training | - Training for non-academic staff no longer available due to national cuts. - Training needs are Laboratory Information Management System; laboratory Quality Management System, Health and Safety; maintaining and repairing equipment, specific methods for analysis - Need to empower laboratory technicians |
| R1B | - No formal training needs assessment - Limited funding for training - High teaching loads of academic staff | - Limited funding for training | - Funding for laboratory technicians only through project funds (rarely happens) - Need for training in Health and Safety, QA, Laboratory Information and Laboratory Management Systems |
| R1C | - High teaching loads of academic staff | - Difference of training opportunities between self-funded and grant-funded students. Self-funded students are sometimes excluded from training grant-funded students receive - No formalised guidelines on identification of training needs - Training needs in thesis/scientific writing, proposal writing, specific software analysis (‘R’, ‘Python’) - Communication about training courses could be more effective (students not aware of all training options) | - Limited funding for training - Need for training in Health and Safety, QA, Laboratory Information and Laboratory Management Systems |
| R1D | - Need for training in critical thinking and critical appraisal skills - High teaching loads | - No formalised guidelines on identification of training needs - Communication about training courses could be more effective (students not aware of all training options) - More in-depth training needed in specific research methods, data analysis software (‘SPSS’, ‘R’), academic and grant writing | - Limited funding for training - Need for training in Health and Safety, QA, Laboratory Information and Laboratory Management Systems |
| R1E | - Training needs in project and financial management | - No formal training needs assessment - Training needs: Endnote | - Training needs not considered the same way as academic staff/students (e.g. lab staff has no opportunity to discuss training needs as there is no line manager) |
| R1F | - Training needs in project and financial management and specific IT training - High teaching loads limit time for training and research | - Difference in access to training between self-funded and grant-funded students - RS students are not aware of the training courses available to them 🡪 need for more effective communication of courses | - Training needs in laboratory information management systems and H&S |
| R2A | - No formal individual training needs assessment - Training needs discussed at meetings are hardly followed up upon - No funding for training at institutional level - Training needs: project and financial management, grant writing, remote sensing, hydro informatics - High teaching loads | - No formal individual training needs assessment - Training needs discussed at meetings are hardly followed up upon - No funding for training at institutional level - Training needs: GIS, statistics, programming software (Python, R) | - No formal individual training needs assessment - Training needs discussed at meetings are hardly followed up upon - No funding for training at institutional level - Training needs: handling of equipment, maintenance, trouble-shooting, and repair - Little in-country expertise in providing such training - Urgent need to empower and consider laboratory technicians in research activities |
| R2B | - No challenges reported | - No challenges reported | - No challenges reported |
| R2C | - No challenges reported | - No challenges reported | - No challenges reported |

### S7_Table 6: Summary of innovative or good practice, and problem solving: learning and teaching

|  | **Suggestions** | **Good practice** |
| --- | --- | --- |
| **Training Academic Staff** | - Ensure effective communication (e.g. online communication) of all training opportunities to academic staff - RS and DFID to meet with HoDs and university administration to sensitise them to the importance of research and to reduce teaching loads of PIs - Develop inventory of trainings across all consortia and open them up to maximise cross-fertilisation - Open up consortia workshops to other students/staff/departments (e.g. advertise through email and departmental meetings) - Develop ‘inventory’ of academic staff skills so that individuals can offer training at the institutional level - Introduce performance management systems, with all staff having line managers with whom they meet formally at least once per year to discuss progress, development and training needs - Establish a programme of continuous professional development with follow-up, linked with a robust learning framework. This could be of greater value for academic and non-academic staff and PGR students and could potentially contribute to institutional learning and growth. This could also be linked to a professional mentoring programme for both staff and PGR students | - Training within department open to all staff and students - Organising writing retreats for academic staff and students to increase research outputs - Quality assurance guidance document on learning and teaching and a Learning and teaching Centre that aims at ensuring teaching and learning excellence in the university - Training for all staff and students is included in annual budgets |
| **Training Students** | - PIs and postgraduate office/graduate school to ensure effective communication (e.g. online communication) of all training opportunities to students - Consider hiring postdoctoral researchers that support both teaching and research activities - ACBI Award Holder Meetings offer suitable platforms to include generic skill training into the overall meeting schedule - Develop inventory of trainings across all consortia and open them up to maximise cross-fertilisation - Open up consortia workshops to other students/staff/departments (e.g. advertise through email and departmental meetings) - Establish formalised, mandatory research methodology course for PhD students - Establish formalised, critical thinking and appraisal skills training - RS training programme needs to be communicated more clearly to PhD students - Need to employ research assistants and postdocs that drive research activities in the department (need to budget for them in research proposals) - Establish a programme of continuous professional development with follow-up, linked with a robust learning framework. This could be of greater value for academic and non-academic staff and PGR students and could potentially contribute to institutional learning and growth. This could also be linked to a professional mentoring programme for both staff and PGR students - Gender awareness/diversity training | - Training within department open to all staff and students - Organising writing retreats for academic staff and students to increase research outputs - Quality assurance guidance document on learning and teaching and a Learning and teaching Centre that aims at ensuring teaching and learning excellence in the university - Revenue from selling laboratory manuals to undergraduate students is reinvested in supporting training for PGR students - Training for all staff and students is included in annual budgets |
| **Training Non-Academic Staff** | - Ensure effective communication (e.g. online communication) of all training opportunities to non-academic staff - Develop inventory of trainings across all consortia and open them up to maximise cross-fertilisation - Include laboratory technicians more effectively in research and departmental activities (e.g. weekly/monthly seminars, acknowledgement in publications, and involvement in drafting funding proposals) - Reflect training needs of non-academic staff more clearly in the budgets of grant proposals - Laboratory technicians attending regional and international training workshops together with students and academic staff to ensure cascading training effect - Open up consortia workshops to other students/staff/departments (e.g. advertise through email and departmental meetings) - Establish a programme of continuous professional development with follow-up, linked with a robust learning framework. This could be of greater value for academic and non-academic staff and PGR students and could potentially contribute to institutional learning and growth. This could also be linked to a professional mentoring programme for both staff and PGR students | - Training within department open to all staff and students - Training for all staff and students included in annual budgets |

### S7_Table 7: Research strategies and support strengths - assessment findings

| **Institution synonym** | **Strategic plans** | **Research support** |
| --- | --- | --- |
| R1A | - Participatory strategic planning meeting for institution, including public and private sectors, and alumni - Main goal to become research centre of excellence and focus on PGR training and research - Focus on entrepreneurship to absorb graduate students - Faculty strategic plan linked to institutional strategic plan | - Research board that provides funding for academic staff to establish themselves as researchers (very limited funding) - Graduate School in place - Research Quality Assurance office and guidelines in place |
| R1B | - Institutional strategic plan in place with focus on entrepreneurship and need to extend collaborations with public and private sector and NGOs - Major aim to improve research profile through increase of PGR students and diversification of funding sources - Department developing own strategic plan | - Doctoral schools in place (different from other institutions) that help promote research agenda and outputs - Effective national and institutional ethics committees in place - Quality Assurance Unit established - Information about research (projects and outcomes) regularly collected and used as evidence of research activity to attract external partners |
| R1C | - University and college strategic plan in place informed by local priorities - College strategic plan includes SWOT analysis and a clear implementation plan | - Research Office offers research and grant administration support services for researchers, including sample grant applications - Institutional repository including intellectual outputs (publications, conference papers, speeches) - School of Graduate Studies responsible for postgraduate admission, progress, examination |
| R1D | - University and college strategic plan in place informed by local priorities | - Research Office offers research and grant administration support services for researchers, including sample grant applications - Institutional repository including intellectual outputs (publications, conference papers, speeches) - School of Graduate Studies responsible for postgraduate admission, progress, examination |
| R1E | - Strategic planning done at the institutional level (strategic plan for 3-year period) | - Research Office provides support for institutional planning, development and funding - Research Office valued by staff and students - Research Office oversees research outputs - Research Office provides funding for conferences and training |
| R1F | - Strategic planning committees at the university, faculty and departmental level with key performance indicators and benchmarking - Intensifying research performance is key strategic area (including increasing PGR enrolment) - Research Committee periodically reviews research themes - Regular progress reports on departmental strategic plan to the faculty - Use of postdoctoral fellows to promote research - Research intensification strategy in place | - Office of research and Development and school of Graduate Studies offer research support - Use of research management software system to track research (applications) record research outputs - Office of research and Development offers ethical and IP support through dedicated units - Competitive funding for emerging researchers from the Office of research and Development - Research Quality Assurance office and guidelines in place - Strong national research commitment through establishment of national academy of science |
| R2A | - Plans for a participatory strategic planning meeting that includes stakeholders from higher education, private and public sector, and NGOs - Inception meeting included stakeholders from public and private sector to ensure that research addresses local needs - RS project addresses need for research in under-researched area, but needed for national development | - Increasing focus on research quality assurance (performance contract with the World Bank) - All departments have records of research projects and activities - Departmental research units and ethics committee in place |
| R2B | - Participatory planning process that is informed by national agenda - Strengthening postgraduate training key strategic objective through the development of new postgraduate courses | - Sufficient financial and administrative support - Administrative division ensures that institutions policies, procedures and regulations are implemented and followed adequately - Technical working group that goes through funding proposals as quality assurance mechanism |
| R2C | - Strategic plans at different levels in place which are reviewed yearly to reflect changes, trends and needs in the market - Strategy is grounded in business theory - Flexible system that is monitored and adjusted regularly | - A lot of different research support in form of different departments providing information about grants, proposals quality assurance mechanism, data base for all research outputs, |

### S7_Table 8: Research strategies and support gaps and challenges - assessment findings

| **Institution Synonym** | **Strategic plans** | **Research support** |
| --- | --- | --- |
| R1A | - Research priority areas more determined by research focus areas of individuals than strategic plans | - Need for a university-wide ethics committee and research support office - Graduate School has limited resources to offer training - Need for more learning from other established graduate schools |
| R1B | - Research priority areas more determined by research focus areas of individuals than strategic plans | - Ethics processes for subjects like geology and hydrology need improvement as they are traditionally did not deal much with research ethics - Need to enhance ethical rigour and awareness about ethics (especially regarding IP and patents) |
| R1C | - No formal departmental strategic plan - Need to establish key research areas to focus research activities and proposal development | - Services of Office of Research and Development not clear to all staff and students (e.g. funding opportunities) - School of Graduate Studies new rules on progression not communicated effectively to PGR students and supervisors |
| R1D | - College strategic plan is currently being updated. Old plan doesn’t include key research priorities - No departmental strategic plan, but departmental board | - Services of Office of Research and Development not clear to all staff and students (e.g. funding opportunities) - School of Graduate Studies new rules on progression not communicated effectively to PGR students and supervisors |
| R1E | - Strategic challenge of retaining researchers/graduates - Departmental strategic plans are not a requirement - Students not included in planning process | - No specific challenges reported |
| R1F | - High teaching loads of academics doesn’t allow for 40% of their time being spent on research, as indicated in the strategic plan - Need for more postdoctoral fellows to promote research in department (but lack of funding) - Strategic challenge of retaining researchers/graduates | - Office of Research and Development could work more effectively with Faculty Deans and Faculty Research Committees - Need to extend awareness around IP |
| R2A | - Outdated strategic plan (>10 years old) - Research priority areas more determined by research focus areas of individuals than strategic plans | - Inadequate research support at university level - Little collaboration between research units - Little technical and administrative support for research - Need to strengthen data management system at university level - Need for more grant proposal development support, as academic staff generally have little experience |
| R2B | - Need to advertise PGR training programmes beyond national borders | - Need to establish a donor intelligence office to assist academic staff to identify funding opportunities and provide donor and funding specific information |
| R2C | - No specific challenges reported | - No specific challenges reported |

### S7_Table 9: Summary of innovative or good practice, and problem solving: research strategy and research support

|  | **Suggestions** | **Good practice** |
| --- | --- | --- |
| **Strategic Plans** | - Students and non-academic staff (e.g. laboratory technicians and support staff) should also be involved in strategic planning activities to ensure needs of all staff included in research and research support are addressed - Consortium partners with effective departmental strategic plans should help less experienced partners through the sharing of resources/examples/good practice - Strategic plan should not cover a period longer than five years - Consider the establishment of doctoral schools to promote research nationally and internationally - In order to address disjoint between institutions’ and governments’ strategic priorities in terms of research, sensitise government to the importance of research | - Participatory strategic planning meetings/workshops, including SWOT analyses and implementation plans with clear performance indicators and predefined targets - Establishment of institutional research committee that reviews research themes periodically to ensure that they are linked to local needs. This can help direct funding efforts and strength research quality assurance mechanisms - Regular departmental reviews conducted by external reviewers to ensure implementation of activities to improve department - Introduction of new requirement for all teaching staff to have PhDs to strengthen research capacity |
| **Research Support** | - Research offices/graduate schools to communicate their services more effectively to academic staff, students and support staff, for instance through regular online communication (e.g. e-newsletters, emails to all staff), organising awareness workshops about research offices/graduate schools and distributing promotion material (e.g. flyers) - Facilitate staff exchanges between research offices/graduate schools across partner institutions within consortia to improve services through knowledge sharing - Research quality assurance documents to be shared across all consortium partners - Establish technical working group to review funding proposals as QA mechanism - Establish a donor intelligence focal point to assist academic staff to identify funding opportunities and provide donor and funding specific information - Create awareness of importance of ethics and IP in science through sharing learning across all consortia (use of all-consortia meetings) - Build in postdoctoral researchers in funding applications who strengthen the research process within departments | - Increased investment in strengthening research quality assurance systems through the establishment of dedicated quality assurance units and the development of quality assurance documents |

### S7_Table 10: PhD programmes strengths - assessment findings

| **Institution synonym** | **Application and registration** | **Supervision and mentoring** | **Progression and examination** |
| --- | --- | --- | --- |
| R1A | - Application and registration process well understood - Comprehensive guidelines for PGR training online - Registration date can be backdated - Student encouraged to apply as known by supervisor | - Supervision guidelines in place - 2-4 supervisors allowed - Skills of supervisors are scrutinised by Faculty Higher Degree Committee - Guidelines state that supervisor and students are required to meet at least 3 time per year (but they normally meet more often) - High emphasis on quality supervision, including frequent supervision trainings - Feedback mechanism for students in place - Good supervisor-student relationship | - Progress monitored regularly as written down in guidance document - Regular progress reports to supervisors - Supervisors regularly report on progress - Student has to present at departmental seminars - Graduate School keeps record of student’s progress - Clear guidelines on examination - Graduate School leads on communication with external examiners - Academic qualifications of examiners scrutinised by Graduate School |
| R1B | - Application and registration process well understood - Transparent student recruitment process | - Voluntary contract between student, supervisor and graduate school - Good supervisor-student relationship - Feedback mechanism for students in place | - Progress monitored regularly - Supervisors have to submit annual progress report - Institution recently tightened rules on PhD completion time - Student has to present at departmental seminars |
| R1C | - PhD handbooks in place | - Good supervisor-student relationship - Institutional supervision guidelines | - Institutional guidelines in place - Progress is monitored regularly and reports are sent back to the Graduate School - Recent policy changes in Graduate School should improve effectiveness - Clear guidelines on consequences if student’s progress is inadequate |
| R1D | - PhD handbooks in place - Formal recruitment process | - Institution provides supervisory training - Good supervisor-student relationship - Increasing engagement of external (national and international) supervisors | - Recent policy changes in Graduate School should improve effectiveness - Monthly departmental seminars to check on PhD students’ progress - Clear guidelines on consequences if student’s progress is inadequate |
| R1E | - Clear application and registration guidelines - Postgraduate handbook in place that is available online - Lab induction for new students in place, includes lab safety training - Formal recruitment process | - Contract between student and supervisors with objectives, timelines and responsibilities 🡪 helps monitoring progress - Good supervisor-student relationship | - Yearly progress report that needs approval from departmental and institutional level - Efficient examination process - New regulation that PhD students need to have at least two publications to pass PhD studies (Master students need one) - Institutional funding for writing retreats |
| R1F | - Clear application and registration guidelines - Postgraduate handbook in place that the students receives upon registration - Increasing interest in postgraduate studies (but lack of funding) | - Recently established supervision guidelines with minimum requirements to be a PhD supervisor (Quality assurance measure) - Good supervisor-student relationship - Mentorship systems in place | - Progress of student formally and regularly assessed - Students needs to submitted regular progress report that are assessed at departmental and institutional level - Informal assessments through regular presentations at the departmental level - Extensions of PhD studies possible, but need approval from Graduate School - Examination process efficient and little challenges with external examiners assessing thesis on time |
| R2A | - Transparent recruitment process | - Supervision and mentoring was discussed at inception workshop - Good supervisor-student relationship - National supervision guidelines in place with requirements for supervisor appointment and supervision training | - Progress monitored through meetings and departmental seminars - PhD students to set up regular skype meetings and Facebook group to check on each other’s progress |
| R2B | - In the process of developing postgraduate handbook | - Good supervisor-student relationship | - Recent policy changes in Graduate School should improve effectiveness |
| R2C | - Clear application/registration process - Induction for new staff in place - Good knowledge of PhD student about other PhD projects | - Good supervisor-student relationship - Student has a supervisor at university and research institution | - Progress of student formally and regularly assessed |

### S7_Table 11. PhD programmes gaps and challenges - assessment findings

| **Institution synonym** | **Application and registration** | **Supervision and mentoring** | **Progression and examination** |
| --- | --- | --- | --- |
| R1A | - Application and registration process lengthy and can take up to 6 months - Difficult for students to pay the fees - Limited knowledge of overall Initiative | - Supervisors normally act as mentors - Need for an external mentor based in UK | - Inconsistent knowledge about deadlines of progress reports |
| R1B | - Little knowledge of overall Initiative and other PhD research projects - Inconsistent knowledge about the availability of PhD guidelines - PhD student only introduced formally towards the end of the inception workshop | - No specific rules on how often student and supervisor should meet - Difficulty to address certain issues (e.g. funding-related) with supervisors | - Funding challenges force students to engage in teaching activities, which prolongs PhD progress |
| R1C | - Need for more formal departmental induction process for PhD students - Long registration process | - Supervision loads too high - No formalised maximum of students to supervise - No supervision guidelines (supervision training not compulsory) - PhD students wondering: ‘Who is supervising the supervisors?’ - Lack of formal feedback mechanisms for students - Process of applying for extension lacks clarity - No requirements on how often supervisory meetings should take place | - Changes of institutional guidelines inadequately communicated to supervisors and students - Significant delays in examination process (up to two years) due to unresponsive examiners, but recent changes in policies should change that - Introduction of teaching cap for PhD students that should help focus on PhD studies |
| R1D | - Long registration process - Need for more formal departmental induction process for PhD students | - Process of applying for extension lacks clarity - High supervision load in combination with high teaching loads - No formalised maximum number of PhD students to supervise - No requirements on how often supervisory meetings should take place - Difficulty to address certain issues (e.g. funding-related) with supervisors | - Significant delays in examination process (up to two years) due to unresponsive examiners, but recent changes in policies should change that |
| R1E | - Need for more formal induction at departmental level - Limited knowledge of overall Initiative | - Lack of support from supervisors beyond RS project - Need for formal mentorship system with students - Existence of mentorship system needs to be communicated more effectively | - No specific challenged reported |
| R1F | - No specific challenges reported | - No specific challenges reported | - Lack of funding for PhD training, which forces many PhD students to take on teaching roles, delaying their progress |
| R2A | - PhD student unaware of PhD handbook - No formal induction process at institutional level | - Lack of clarity on how national supervision guidelines are implemented at institutional level | - Inconsistent knowledge on how PhD progress is monitored |
| R2B | - Lack of clarity whether there was a formal application process (PhD student very experienced and worked with PI before) - Long registration process - Need for more formal departmental induction process for PhD students | - Neither formalised requirements on how often PhD student and supervisor should meet nor supervision guidelines | - No specific challenges reported |
| R2C | - No knowledge/information about whether laptop is part of the stipend | - No specific challenges reported | - No specific challenges reported |

### S7_Table 12: Summary of innovative or good practice, and problem solving: PhD programmes

|  | **Suggestions** | **Good practice** |
| --- | --- | --- |
| **Application & Registration** | - Develop PhD specific inductions at institutional/departmental level. Inductions as peer-to-peer activities involving more senior PhD students - Funders to develop clear guidelines and criteria for the selection process (e.g. how PhD scholarship is advertised, selection process, criteria for candidates) - Ensure that Scheme notes and proposal, including the PhD projects, is communicated effectively to the PhD students and the whole research team | - Provision of PhD handbook and guidelines online |
| **Supervision & Mentoring** | - Rolling out supervision contracts between supervisors and students across all partners of a consortium - Use consortium meetings to develop gender-sensitive supervision/mentorship systems in a participatory way - Use consortia workshops to discuss supervision, mentoring and progress of PhD students. Circulate workshop meetings notes to formalise rules and guidelines discussed in meetings | - Increase the number of PhD qualified staff across all institutions, for instance through new policies requiring all academic staff, including supervisors of PhD students, to have a PhD - Student-supervisor contracts that include clear roles and responsibilities, targets and objectives with times lines, training schedules and meeting dates - Having female mentor in the UK as an external feedback mechanism |
| **Progression & Examination** | - Establish PhD monitoring committees within departments that monitor students’ progress - Roll out of peer-to-peer progress monitoring system across all consortia | - Review and tighten policies on PhD progression and extension, e.g. inform the Graduate School immediately if student encounters something that influences progress, instead of waiting until the annual progress report or asking for an extension at the end of the programme - Set-up of peer-to-peer progress monitoring system, including regular *Skype* calls and *WhatsApp* groups to check on each other’s progress - Offering honorarium or backlisting external examiners as mechanisms to make examination process more efficient - Publishing in international peer-reviewed journals as a requirement to pass PhD in order to strengthen individual and institutional research capacity |

### S7_Table 13: Financial management and funding strengths - assessment findings

| **Institution synonym** | **Financial management** | **Funding** | **Financial sustainability** |
| --- | --- | --- | --- |
| R1A | - Bursar’s department with many sub-divisions, one deals with research grants - Clear and strict accounting regulations - Overheads go to the university and are between 3-5% | - National Research Council in place that has limited funding - Limited competitive funding at the institutional level - RUFORUM critical research partner in terms of funding - Focus on diversify institutional funding regime - Lack of clarity among ACBI students with regard to funding | - Focus on establishing new national and international collaborations - Focus on commercialising research technologies and getting patents |
| R1B | - Each research project has project account in line with funder’s requirements - Projects with international funding have been gradually centralised in recent years - Overheads go to the university and are between 2-10% depending on terms and conditions of project - Strict and clear guidelines based on national policies - Experienced financial staff that is used to deal with large international grants | - Majority of funding comes from teaching - Majority of funding for research comes from external funders - Ministry of Higher Education provided limited start-up grants for researchers - World Bank funding for research capacity strengthening | - Focus on establishing new national and international collaborations - Collaboration of Doctoral Schools crucial to attract national and international funders |
| R1C | - Finance office ensures funds are managed in line with funders’ regulations - Financial management system considered largely effective | - No particular strengths reported | - Identifying departmental/institutional research priority areas to streamline funding efforts - Focus on establishing new national and international collaborations |
| R1D | - Financial management system considered largely effective | - No particular strengths reported | - Promotion of laboratory services to generate income - Focus on establishing new national and international collaborations |
| R1E | - Projects funds managed by Research Office - Specific project accounts - Financial management system considered largely effective | - National funds for research and postgraduate training available - Institutional funds for research and postgraduate training available | - No particular strengths reported |
| R1F | - Grant department in charge of financial management of grants - Specific project accounts - Financial management system considered largely effective due to strict guidelines | - Government sponsors undergraduates and postgraduate diplomas - Institutional research start-up grants | - Intensified collaboration between Research Office and Graduate School seeking government and industry support for research - Offering laboratory services to external partners - Selling laboratory manuals |
| R2A | - Financial management systems described as fit for purpose, finance office overseeing financial management | - No specific strengths reported | - No specific strengths reported |
| R2B | - Financial management system considered largely effective due to strict guidelines | - Good track record of generating funding from external partners | - Core academic staff requested to bring in funding |
| R2C | - Financial management system considered largely effective due to strict guidelines | - Sustainable funding streams from government and international funders | - Nationally and internationally very well renowned - Sustainable funding streams from government and international funders |

### S7_Table 14. Financial management and funding gaps and challenges – assessment findings

| **Institution synonym** | **Financial management** | **Funding** | **Financial sustainability** |
| --- | --- | --- | --- |
| R1A | - PIs are largely responsible for financial management of own grants | - Limited governmental funding for research and postgraduate training - Current socio-economic situation makes funding situation more challenging - Lack of funding for equipment - Need for funding for Graduate School to offer training | - Commercialisation of research is not always welcome by academics - Limited time of researchers to apply for international research funds |
| R1B | - No specific challenges reported | - Limited governmental funding for research - Currently no institutional funding for research available - Individuals in departments lead research agenda - Lack of clarity among ACBI students with regard to funding | - Limited time of researchers to apply for international research funds |
| R1C | - PIs would like to receive monthly financial reports from finance office - No finance/admin support at departmental level - Unclear how overheads are used across university, college and departments - PIs are largely responsible for financial management of own grants | - Huge difference between grant and self-funded students - Lack of clarity among ACBI students with regard to funding - Relatively undiversified funding regimes | - Limited time of researchers to apply for international research funds |
| R1D | - PIs would like to receive monthly financial reports from finance office - No finance/admin support at departmental level - Unclear how overheads are used across university, college and departments - PIs are largely responsible for financial management of own grants | - Huge difference between grant and self-funded student - Lack of clarity among ACBI students with regard to funding - Relatively undiversified funding regimes | - Limited time of researchers to apply for international research funds |
| R1E | - No specific challenges reported | - Systems for keeping students informed about their level of funding may benefit from review to ensure it meets the needs of the students - Lack of clarity among ACBI students with regard to funding | - No specific challenges reported |
| R1F | - There is no admin/finance support at departmental level - PIs are responsible for financial management of own grants | - Government does not support doctoral studies - Government’s low prioritisation of research - Limited institutional funds for research and equipment - Need to diversity funding regimes - Lack of clarity among ACBI students with regard to funding | - Limited time of researchers to apply for international research funds - Laboratory capacity strengthening would attract more postgraduate students, which generates income |
| R2A | - PIs are largely responsible for financial management of own grants | - Lack of national and institutional funds for research - Socio-economic challenges in the country led to universities not receiving their full budget for teaching and research - Limited support from government and institution for doctoral studies | - Need to strengthen collaborations to intensify research |
| R2B | - No specific challenges reported | - Little governmental funding for research | - Need to diversify funding regime due to government spending cuts that affect research |
| R2C | - No specific challenges reported | - No specific challenges reported | - No specific challenges reported |

### S7_Table 15: Summary of innovative or good practice, and problem solving: Financial management and funding

|  | **Suggestions** | **Good practice** |
| --- | --- | --- |
| **Financial Management** | - Developing clear institutional policies on the use of overheads and communicate them effectively to all staff. Such policies help ensure to maximise revenue for research - Create transparency as to the amount and use of funds from project overheads at the departmental level. Prioritise the use of these funds in line with a departmental level plan and establish a system for assessing them - Establish regular reporting from finance offices to PIs - Facilitation of financial staff exchange visits where staff with inefficient systems learn from more efficient institutions - Where possible, include funds for administrative/finance support staff in grant proposals - PIs to share experiences of financial management with each other, for instance, at Award Holder Meetings in Africa and the UK |  |
| **Funding** | - Developing clear institutional guidelines on recommended amount of PhD stipends - Ensure open communication about funding for PhD students - Identify international collaborators and share among department/institution to maximise funding opportunities and efforts - Consider focussing on research priority areas within departments - Consider marketing laboratory services (e.g. renting out equipment and space) - Development of research intensification strategies through close partnerships with national policy makers, universities, private sector and NGOs - Use ACBI links efficiently to maximise collaborations - Discussing funding challenges with regard to devaluation of Pounds since Brexit at Award holder meetings - Internationalisation and diversifying funding regimes to bring about more sustainable funding for research and strengthening the research capacity more generally (e.g. also in terms of human resources) | - Research Office developing guidelines on PhD stipends - Opening up consortia (inception) workshops/meetings to broader audiences (including other departments, faculties, public sector, private sector, NGO, research institutes) for networking purposes to build partnerships important for diversifying funding regimes |

### S7_Table 16: Collaboration and partnerships strengths - assessment findings

| **Institution synonym** | **Current collaborations and partnerships** | **Future plans** |
| --- | --- | --- |
| R1A | - Strong strategic focus on building research collaborations and partnerships with industry (national and international), government, NGOs, research institutes - Office for Information and International Relations responsible for establishing and maintaining partnerships   - Current focus building strong relationship with industry (through planned placements) to enable graduates to be absorbed in the labour market - Large research consortia/programmes (e.g. ACBI, RUFORUM) crucial with regard to partnership - Increase collaboration with international universities to promote knowledge exchange and get more expertise - Some laboratory services are used by commercial farmers | - Faculty currently establishing an ‘institute’ that focuses on partnering with industry - Organising workshops with partners from industry to discuss collaborations - ACBI crucial to expand collaborations and partnerships internationally |
| R1B | - Increasing focus on building research collaborations and partnerships with industry (national and international), government, NGOs, research institutes - Doctoral Schools have excellent research collaborations and partnerships with different stakeholders through being part of research consortia - Inception workshop included many stakeholders from different institutions - (Little) funding for academic staff exchange to strengthen capacity and research networks - Focus on internal collaboration | - ACBI crucial to expand collaborations and partnerships internationally |
| R1C | - Strategic focus on building research collaborations and partnerships with industry (national and international), government, NGOs, research institutes | - ACBI crucial to expand collaborations and partnerships internationally |
| R1D | - Strong strategic focus on building research collaborations and partnerships with industry (national and international), government, NGOs, research institutes | - ACBI crucial to expand collaborations and partnerships internationally |
| R1E | - Strong strategic focus on building research collaborations and partnerships with industry (national and international), government, NGOs, research institutes - Dedicated International Linkages Office responsible for strengthening research capacity, staff exchange, scholarly visits, mentorship for staff interested in exchange visits | - ACBI crucial to expand collaborations and partnerships internationally |
| R1F | - Strong strategic focus on building research collaborations and partnerships with industry (national and international), government, NGOs, research institutes - Policy on Internationalisation, which includes objective of expanding international research cooperation   - Recruiting visiting researchers   - Cooperative supervision   - Encourage international students to participate in research at the institution   - Presenting research at international conferences/workshops, etc. - Focus on increasing collaborations in Africa 🡪 ACBI therefore crucial - Focus on internal collaboration - Conducting laboratory awareness workshop to promote laboratory services to industry - Departmental policy on enhancing collaborations with industry | - Continued focus to increase partnerships and collaboration |
| R2A | - Strong existing international research partnerships | - ACBI crucial to expand collaborations and partnerships internationally |
| R2B | - Maintaining strong linkages with local and international stakeholders. Benefits:   - Sharing costs of research   - Build capacity of staff   - Exchange research material at low cost   - Strengthen research environment - North-South and South-South collaborations are key - Focus on strengthening links with francophone African countries - Strong links with industry - ‘Firm based approach’: finding out from industry what their research needs are | - ACBI crucial to expand collaborations and partnerships internationally |
| R2C | - Maintaining strong linkages with local and international stakeholders - Strong links with industries | - ACBI crucial to expand collaborations and partnerships internationally |

### S7_Table 17: Collaboration and partnerships gaps and challenges - assessment findings

| **Institution synonym** | **Current collaborations and partnerships** | **Future plans** |
| --- | --- | --- |
| R1A | - Difficulty of building partnerships from industry and policy makers - ‘unwillingness’ of these stakeholders to use research to inform their work - Need to build laboratory networking/collaborating mechanism | - Need for more award holder meetings |
| R1B | - Need to further strengthen laboratory networking | - Lack of knowledge within the department of the ACBI which might be detrimental to partnership building |
| R1C | - Difficulty of building partnerships from industry and policy makers - Need to build laboratory networking/collaborating mechanism | - Lack of knowledge within the department of the ACBI which might be detrimental to partnership building |
| R1D | - Difficulty of building partnerships from industry and policy makers - Need to build laboratory networking/collaborating mechanism - Difficulty for junior researchers to identify research collaborators | - No specific challenges reported |
| R1E | - Research partnerships need a lot of resources (time, commitment) 🡪 need to carefully consider benefits of partnerships - Difficulty to interact and collaborate between/within departments/faculty | - Lack of knowledge within the department of the ACBI which might be detrimental to partnership building - Need for more award holder meetings |
| R1F | - Difficulty to interact and collaborate between/within departments/faculty - Need to promote research e.g. through newsletters and workshops | - Lack of knowledge within the department of the ACBI which might be detrimental to partnership building - Need for more award holder meetings |
| R2A | - Difficulty of building partnerships with non-francophone partners   - ACBI therefore crucial - Staff’s limited involvement in collaborative research - Little experience of writing collaborative proposals - Little knowledge on international grants | - No specific challenges reported |
| R2B | - No specific challenges reported | - No specific challenges reported |
| R2C | - No specific challenges reported | - No specific challenges reported |

### S7_Table 18: Summary of innovative or good practice, and problem-solving examples: research collaborations and partnerships

|  | **Suggestions** | **Good practice** |
| --- | --- | --- |
| **Research Collaborations & Partnerships** | - Development of strategies to increase South- South collaborations especially with countries that have different official language (e.g. Francophone, Anglophone) - organising research awareness workshops for external stakeholders (e.g. industry, policy makers), increased cross-consortia collaboration, share learning and best practice about successful research partnerships (e.g. ‘how to guide’) - Develop more effective communication around ACBI and other research projects within and across departments in order to establish research links and facilitate cross-fertilisation of the Initiative and projects - Laboratories to share their learning and good practice examples across consortia, for instance, at Award Holder Meetings or through online communication (e.g. flyers, newsletters) - Establish clear communication plan within consortia to create equitable partnership - Considering setting up Doctoral Schools who can drive strategic research area and strengthen national and international research collaborations | - Invite policy makers, industry, NGO other research institutions to inception workshop to ensure research is relevant to local needs - Establishment of specific offices/departments in place to increase international partnerships - Development of departmental newsletters about current research shared within and across departments - Development of institutional internationalisation policy - Increased international staff exchanges and better visibility at international conferences as mechanisms to increase national and international research collaborations and partnerships - Development of departmental newsletters that are shared with other departments to provide information on the type of research conducted 🡪 increase of inter-departmental collaborations |
